# Unique Residues Involved in Activation of the Multitasking Protease/Chaperone HtrA from *Chlamydia trachomatis*

**DOI:** 10.1371/journal.pone.0024547

**Published:** 2011-09-08

**Authors:** Wilhelmina M. Huston, Joel D. A. Tyndall, William B. Lott, Scott H. Stansfield, Peter Timms

**Affiliations:** 1 Institute of Health and Biomedical Innovation, Queensland University of Technology, Kelvin Grove, Queensland, Australia; 2 School of Pharmacy, University of Otago, Dunedin, New Zealand; Stanford University, United States of America

## Abstract

DegP, a member of the HtrA family of proteins, conducts critical bacterial protein quality control by both chaperone and proteolysis activities. The regulatory mechanisms controlling these two distinct activities, however, are unknown. DegP activation is known to involve a unique mechanism of allosteric binding, conformational changes and oligomer formation. We have uncovered a novel role for the residues at the PDZ1:protease interface in oligomer formation specifically for chaperone substrates of *Chlamydia trachomatis* HtrA (DegP homolog). We have demonstrated that CtHtrA proteolysis could be activated by allosteric binding and oligomer formation. The PDZ1 activator cleft was required for the activation and oligomer formation. However, unique to CtHtrA was the critical role for residues at the PDZ1:protease interface in oligomer formation when the activator was an *in vitro* chaperone substrate. Furthermore, a potential *in vivo* chaperone substrate, the major outer membrane protein (MOMP) from *Chlamydia*, was able to activate CtHtrA and induce oligomer formation. Therefore, we have revealed novel residues involved in the activation of CtHtrA which are likely to have important *in vivo* implications for outer membrane protein assembly.

## Introduction

*Chlamydia (C.) trachomatis* is the etiological agent for the most prevalent bacterial sexually transmitted infection world-wide. The microorganism is an obligate intracellular bacterium, which is estimated to have diverged to the intracellular niche from a common ancestor some 750 million years ago [1]. Consequently, the *chlamydiae* have evolved a reduced genome of around 1000 protein coding genes, which has resulted in novel modifications of conserved bacterial proteins [2]. *C. trachomatis* HtrA (CtHtrA), the chlamydial ortholog of *Escherichia* (*E.*) *coli* DegP [3], [4], is a member of the HtrA (High temperature requirement protein A) protease family, which are widely conserved among single and multicellular organisms [5]. In well characterised bacterial systems, DegP has been shown to be essential for virulence, and has been implicated in virulence factor secretion such as the filamentous haemagglutinin of *Bordetella pertussis* [6]. CtHtrA is upregulated during *Chlamydia* persistence disease models and stress conditions [4], [7], which is consistent with an important role in the *Chlamydia* life cycle. Whether CtHtrA is essential for *Chlamydia* viability and virulence has not been definitively established, however, as there are currently no molecular tools to generate gene deletions or complementations in *Chlamydia*. As with DegP in other bacterial systems, CtHtrA is an attractive target for antimicrobial drug design.

DegP conducts bacterial stress responses and virulence functions that are specifically associated with protein quality control [8], [9] (reviewed, [10]). While the DegP protease activity recognises and degrades misfolded and mislocalised proteins, the chaperone activity promotes correct folding or refolding of denatured proteins, prevents unwanted aggregation and protects outer membrane proteins from degradation by other proteases during their passage through the periplasm [11]. The ability to conduct both proteolysis and chaperone activity to maintain protein quality ensures this protein is often essential for bacterial pathogenesis [12], [13].

DegP proteases utilise a unique mechanism of allosteric regulation that involves both conformational and oligomeric changes [14]. All members of this family have a serine protease domain with a chymotrypsin fold (S01) in the PA clan (MEROPs database) [15] and either one or two C-terminal PDZ (PSD-95/Dics-Large/ZO-1) domains [16]. The mechanism of DegP proteolysis activation involves a cascade of allosteric activation, conformational rearrangement, and oligomerisation. The protein ‘rests’ as an inactive hexamer, in which the proteolytic active site of each monomer is occluded by the extended LA loop of an opposite monomer [17]. Unfolded protein substrates or small hydrophobic peptides bind to the activator cleft in PDZ1 and induce rearrangement of the protease to a proteolytically active trimer. Structural rearrangements involved in activation include loop rearrangement surrounding the active site and subsequent oligomerisation of the trimer to proteolytically active 12-mer and 24-mer complexes [18], [19]. Assembly into large oligomers is also thought to be the mechanism by which DegP is able to chaperone ß-barrel proteins in their fully folded conformation during transport to the outer membrane in Gram negative bacteria [20].

The ability to differentially respond to the substrates which require either chaperone or proteolysis activity is one of the hallmarks of this unique protein. Yet, the DegP components which control and mediate between these two distinct functions remain to be identified. In this work we demonstrated distinct oligomeric responses when CtHtrA proteins with mutations in either the PDZ1 activator cleft or the PDZ1:protease interface were activated. These distinctions were dependent on whether the activator was a peptide or a protein chaperone substrate.

## Results

### CtHtrA prefers to cleave sequences with non-polar residues at P1

**Figure 1:**
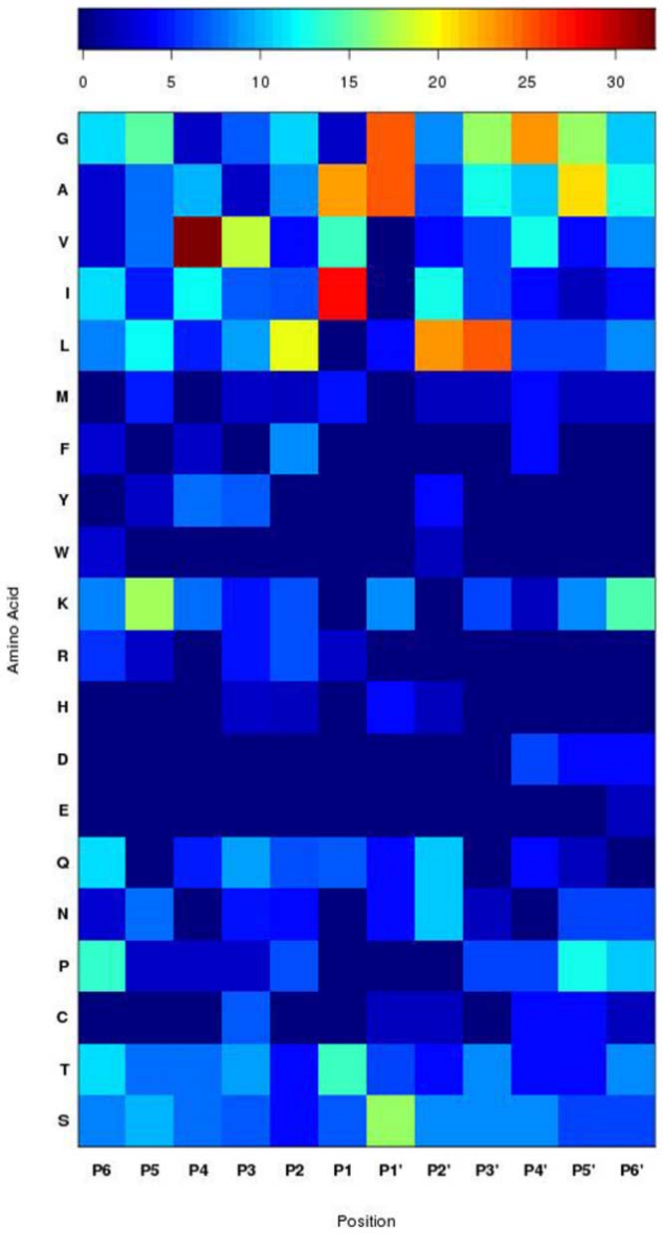
Heat map of CtHtrA peptide cleavage sites identified by PICS. Summary of all PICS data (raw data is shown in Fig. S2). The colour key is indicated below the figure. The relative position in the peptides is indicated at the top and each residue is listed to the left.

To analyse the substrate sequence specificity, CtHtrA was screened against two peptide libraries and then kinetic analysis was conducted against specific peptide sequences. Initial CtHtrA proteolysis screening against an endopeptidase derived GluC PICS peptide library [21] identified a total of 85 cleavable peptide sequences, with a preference for non-polar amino acids on both the prime and non-prime sites (Fig. 1, Fig. S1A, and Fig. S1B). Analyses using the GluC library illustrated a preference for I/A/V in P1 and G/A in P1′. No peptides with L at P1 were identified although L was frequently identified at P2′ and P3′. L was most frequently identified at P2, although there was a greater variety of residues identified at P2 compared to P2′ and P3′.

To further refine the CtHtrA substrate specificity, the REPLi peptide library (Mimotopes, Melbourne) was screened. The REPLi library peptides consist of a randomised tripeptide sequence flanked on either side by two GGG tripeptide sequences. The N-terminus of each peptide was labelled with a 7-methoxycoumarin (MCA) fluorophore, and the C-terminus was labelled with a 2,4-dinitrophenyl (DNP) fluorescent quencher. The peptides were pooled according to structural similarity and selected wells with high activity were then rescreened as individual peptides to identify the peptides cut by CtHtrA (Fig. S2, all wells where CtHtrA activity was observed). The cleaved peptides always had at least one hydrophobic residue (I, L, A, V, or P) in the variant trimer sequence (Table 1). Cleavage at the glycine or terminal fluorophore or quenchers was never detected for any peptide. Where peptides had either an I or L at the same site in the variant sequence, there was always higher cleavage rates with I.

**Table 1:** Peptide sets tested from selected positive wells REPLi library.

Peptides^1^	Δfluorescence^2^	Peptides^1^	Δfluorescence^2^
LLA	0	IAF	1.344 (0.14)
LLV	1.9 (0.10)	IAY	0.41 (0.043)
ISF	3.9 (0.30)	IVF	0.19 (0.016)
ISY	2.84 (0.06)	IVY	0
ITF	0	LAF	0
ITY	0	LAY	0
LSF	0	LVF	0
LSY	0	LVY	0
LTF	0	ASF	0.282 (0.029)
LTY	0	ASY	0
IIA	0.6 (0.02)	ATF	0
IIV	0	ATY	0
ILA	0.403 (0.07)	VSF	0.92 (0.043)
ILV	2.2 (0.03)	VSY	1.77 (0.099)
IFA	0.41 (0.09)	VTF	0.263 (0.016)
IFV	0.6 (0.08)	VTY	0
IYA	0		
IYV	0		

1 Each peptide consists of a variant trimer (as listed in the column) between consistent trimers of glycine with a flourophore and quencher (MCA-GGG XXX GGG-DNP).

2 min^−1^ µg CtHtrA^−1^ mean and (standard deviation).

A series of peptide substrates were then designed based on the cleavage sites previously identified in ß-casein [3], PICS results, previously published substrates for *E. coli* DegP [22], and the REPLi library screening results. These peptides were assayed against CtHtrA and kinetic analysis was conducted (Table 2). The ‘best’ CtHtrA substrate was MFKLI-pNA (Kcat/Km = 1084.8). The data suggests that I was the most preferred residue in the P1 site, with V and L the next preferred residues for P1. However the activity does not depend solely on the residue at P1 with at least P2-P4 also influencing the activity. As the most preferred P1 residues; V, T, and I all have beta-methyl groups, this also suggests an unusual preference for beta-branched amino acids at P1 site. Generally, peptides with only 4 residues or less were not cleaved. FKLI-pNA was the only four residue peptide that was cleavable by CtHtrA. The preference for K at P3 implies a salt bridge may be involved in substrate coordination in the CtHtrA active site in the substrate pocket or subsite S3. In a direct comparison between MFRLI-pNA and MFQLI-pNA, R was clearly the most preferred. MFRLI-pNa exhibited a ten-fold higher Vmax, which is consistent with the residue at the P3 site being involved in a salt bridge that is important for substrate binding. The Km for each of these, however, was at least an order of magnitude higher than the Km for MFKLI-pNa. The results from individually synthesised peptides (Table 2) and the peptides sourced from the REPLi library (Table 1) were consistent in that non-polar sequences are most preferred. MCA-IRRVSYSF-DNP, a peptide based on the optimal activity for human HtrA2 [23] was not a viable substrate for CtHtrA (data not shown). Two cleavage sites were observed in the peptide based on a ß-casein cleavage site (MCA-ENLHLPLPIIF-DNP, **Bcas1**) when analysed by LC-MS-MS (MCA-ENLH↓LPLPI↓IF-DNP), one was not a site previously identified in the full length ß-casein assay [3] (Fig. S3).

**Table 2:** Peptide substrates of CtHtrA.

Substrate	Source of peptide	Vmax (mM sec^−1^, µM CtHtrA^−1^)	Km (mM)	Kcat/Km (M^−1^ s^−1^)
DPMFKLV-pNA	*E. coli* substrate [22]	4.678	0.0003233	241.2
DPMFKLP-pNA	Model *E. coli* substrateP at P1	2.917	0.003874	159.8
DPMFKLL-pNA	Model *E. coli* substrateL at P1	3.179	0.0008829	763.3
DPMFKLI-pNA	Model *E. coli* substrateI at P1	10.340	0.0005271	32.7
PMFKLI-pNA	1 residue less	9.931	0.0003142	526.7
MFKLI-pNA	2 residues less	4.046	0.00006204	1084.8
FKLI-pNA	3 residues less	0.9723	0.0005740	28.2
DMPFKLT-pNA	Model *E. coli* substrateT at PI	8.955	0.0009404	158.7
DPMFKQT-pNA	Model *E. coli* substratechange P1 and P2	0.3165	0.000009381	542.2
MFRLI-pNA	P3 modification	18.53	0.002908	106.22
MFQLI-pNA	P3 modification	0.1796	0.0001409	21.2

### CtHtrA protease activity can be increased in rate by the presence of an activator peptide

**Figure 2:**
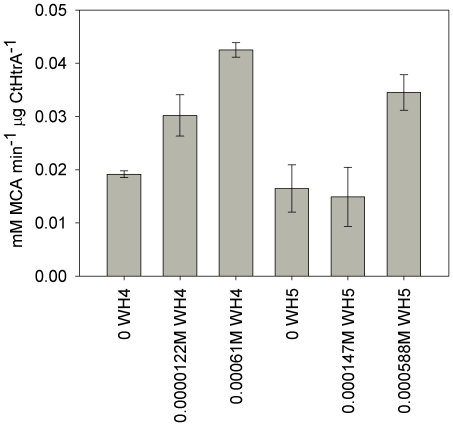
CtHtrA protease activity can be activated by ß-casein C-terminal based peptide (Act1) to greater rates than insulin b-chain C-terminal based peptide (Act2). Rates of generation of fluorophore (or cleavage of **Bcas1** peptide releasing fluorophore from quencher) per minute per µg of CtHtrA (y-axis) are higher for **Act1**. The activators tested were **Act1** (NH_2_-VLGPVRGPFPIIV-OH) based on the C-terminal sequence of β-casein and **Act2** (NH_2_-CGELGFFYTP-OH) based on the C-terminal sequence of insulin (b-chain), with different concentrations compared to the rate without any activator added (x-axis).

**Figure 3:**
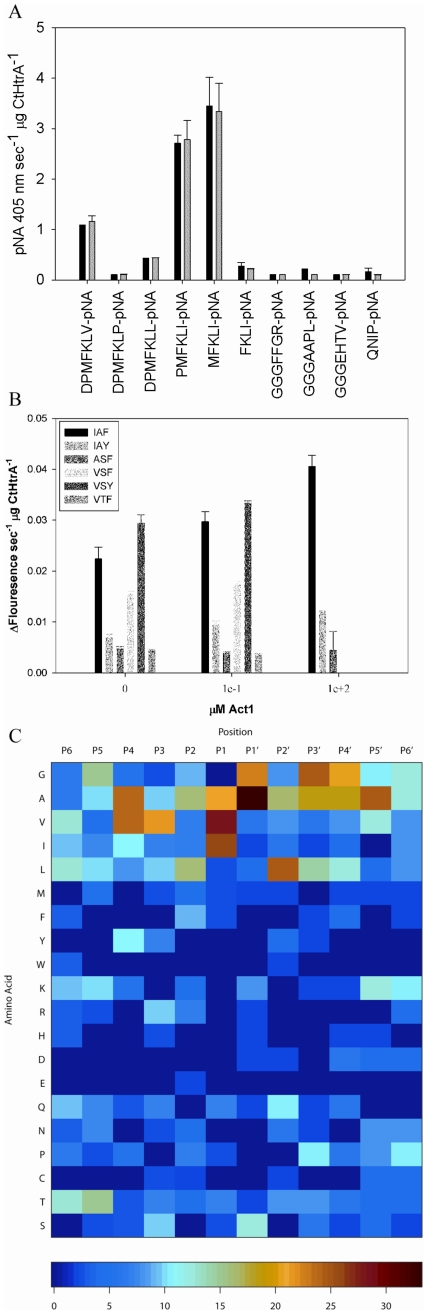
Activation of CtHtrA cleavage of smaller pNA based peptides by Act1 activator. A. pNA substrate cleavage represented by the change in absorbance at 405 nm per µg CtHtrA per second (y-axis). The substrates were tested with **Act1** (NH_2_-VLGPVRGPFPIIV-OH 120 µM; grey) or without **Act1** (black bar). B. Activation of CtHtrA cleavage of flourimetric peptides by **Act1**. The Fig. shows the rate of increase of fluorescence per µg CtHtrA (y-axis) for each of the peptides (left to right: IAF, IAY, ASF, VSY, VTF), at 0, 12.2 µM Act1, and 120 µM **Act1**. C. Heat map of peptide cleavage sites of CtHtrA as identified by PICS in the presence of the Activator peptide (120 µM). The figure is a summary of all the data in Fig. S4. The colour key is indicated to the bottom of the figure. The relative position in the peptides is indicated at the top and each residue is listed on the left side of the figure.

To determine if the addition of a second peptide to the protease assays could activate or increase CtHtrA proteolysis, two activator peptides were tested in the CtHtrA cleavage of the model ß-casein peptide substrate **Bcas1**. The first was based on the ß-casein C-terminal sequence (**Act1**: NH_2_-VLGPVRGPFPIIV-OH) and the second on insulin b chain C-terminal sequence (**Act2**: NH_2_-CGELGFFYTP-OH). Both of these proteins are known to be *in vitro* substrates of CtHtrA, hence their C-terminal sequences may be involved in activation of CtHtrA [3]. The ß-casein C-terminal based activator peptide (**Act1**) showed the greater ability of the two activators to increase the proteolysis rate (Fig. 2). Following this result, the proteolysis rates of other known CtHtrA substrates was then monitored in the presence of **Act1**. Not all substrates were cleaved at a faster rate in the presence of the activator peptide (Fig. 3); and in general, the pNA substrates showed no difference in rate. No evidence of **Act1** cleavage by CtHtrA could be detected when examined by MALDI MS MS (data not shown).

One explanation for the observation of a lack of activation for some substrates could be that these substrates act as both activators and substrates by binding to both the active and allosteric sites of CtHtrA. In particular, the pNA peptides were not activated, which could imply that the pNA moiety itself may bind at the activation site. In order to test this possibility, a peptide (QNIPF) with an F which has as similar structure to the pNA but lacks the nitro-group, was tested as an activator. This peptide activator did not activate CtHtrA cleavage of **Bcas1** but did activate the cleavage of some of the pNA peptides tested (data not shown). This supports the hypothesis that at least some pNA substrates can act as both activators and substrates.

To assess if the presence of the activator peptide alters the substrate specificity, the role of the **Act1** activator was further examined using PICS library screening. The addition of the activating peptide increased the observed frequency of V and I in P1 and A in P1′ (Fig. 3), but did not alter the specificity. The presence of the activating peptide increased the number of identified peptides (90) compared to the non-activating peptide assay (85) (Fig. S4A and Fig. S4B).

### CtHtrA PDZ1 carboxyl terminal binding cleft has a role in activation and substrate specificity

**Figure 4:**
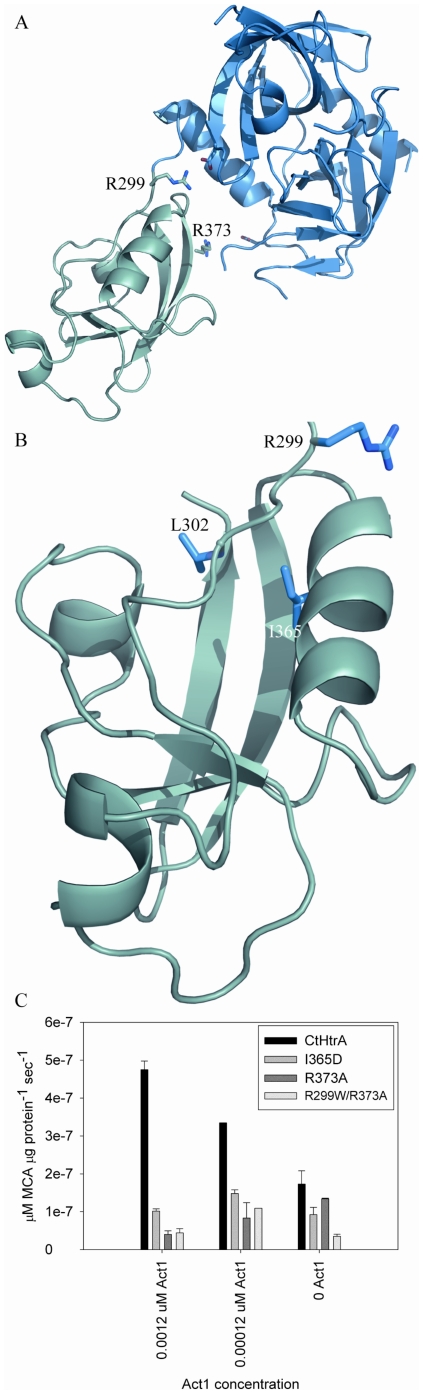
The PDZ domain functions in CtHtrA activation by Act1. A. Modelled PDZ- protease domain (dark blue) interface showing the conserved salt bridge via R299 – D169 and a putative stabilizing salt bridge via R373 – D193. B. PDZ1 putative activator cleft showing residues R299, L302 and I365. C. Activity of CtHtrA wild-type, I365D, R373A and R299W/R373A mutants against **Bcas1** in the presence (grey; 120 µM) and absence of **Act1** (black).

The hexameric CtHtrA molecular model was examined to identify the putative PDZ1 activator binding cleft (Fig. 4). The base of this cleft in CtHtrA is a hydrophobic pocket formed by the residues L302 and I365 (V328 in *E. coli* DegP) (Fig. 4). L302 and I365 were targeted for site directed mutagenesis (L302N and I365D) to make this non-polar pocket either polar or charged. The role of a potential conformational change caused by movement of the whole PDZ1 domain relative to the protease subunit was also tested by mutating a highly conserved arginine residue (R299W) on the flexible loop between the protease and PDZ domains [24]. This mutation was made in order to alter the flexibility around this residue while occupying a similar sized area. Additionally, a second mutation was made in conjunction with the R299W mutation on the residue R373A which potentially forms a salt bridge between the PDZ domain and protease domain. This double mutation was made in order to reduce the flexibility and disrupt the mobile salt bridge involved in the PDZ1:protease domain interface. The R373A mutation was also made independently of R299W. These mutated proteins were assayed to examine the impact of the changes on **Act1** activation on the **Bcas1** substrate proteolysis. The R299W and L302N mutations drastically reduced the protease activity on **Bcas1** to below the limits of detection in this assay. The I365D, R373A, and R299W/R373A mutations all retained the ability to cleave **Bcas1**, although at a markedly reduced rate compared to the CtHtrA wild-type (Fig. 4C). Interestingly, while R299W was inactive in this assay; the activity was somewhat restored by the R299W/R373A combined mutation. **Act1** failed to increase the proteolysis rate for any of these three mutated proteins, indicating that these residues are involved in the activation of proteolysis. The mutated proteins were tested for protease activity against ß-casein. The L302N CtHtrA mutant was unable to cleave ß-casein even after two hours incubation, while the R299W, R3783A, I365D, R373A, and R299W/R373A mutants were all active (data not shown).

### CtHtrA is able to form large 24-mer multimeric complexes which are impacted by mutations in the activator cleft

**Figure 5:**
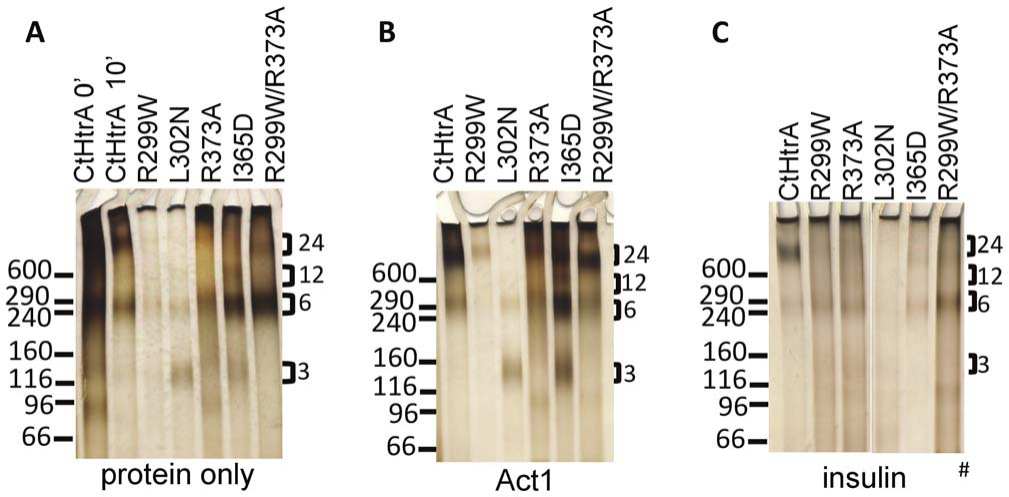
CtHtrA is activated to form 24-mer multimeric complexes which are impacted by mutations in the PDZ1 domain. The figure shows glutaraldehyde crosslinked protein samples prepared by oligomerisation assays which have been separated on 3–8% TrisAcetate gels prior to silver staining. The different multimeric forms of the protein are indicated to the right and the molecular weights indicated on the left (High molecular weight marker, Invitrogen). A. (protein only): 1. CtHtrA 0′, 2. CtHtrA 10′, 3. R299W 10′, 4. L302N 10′, 5. R373A 10′, 6. I365D 10′, 7. R299W/R373A 10′. B. Protein with **Act1** 10′: 1. CtHtrA, 2. R299W, 3. L302N, 4. R373A, 5. I365D, 6. R299W/R373A. C. Protein with **Insulin** 10′: 1. CtHtrA, 2. R299W, 3. R373A, 4. L302N, 5. I365D, 6. R299W/R373A. # This picture is constructed from a single gel, only that the molecular weight marker lane has been removed from the middle of the gel image for consistency with the other gels.

*E. coli* DegP proteolysis activation involves the transition from resting hexamers via trimers to 12- and 24-mers following the peptide activator binding to the PDZ1 binding cleft [18]. CtHtrA oligomer formation induced by **Act1** addition was examined after incubation for 10 mins at 37°C. The wild type and mutant proteins were incubated with either the ß-casein based activator peptide (**Act1**), or with whole insulin (intact), or in the absence of any activator as a control. CtHtrA incubated in the absence of any added activators formed 24-mers with some hexamers detected after 10 mins (Fig. 5). The incubation of CtHtrA with **Act1** induced the protein to form 24-mers after 10 mins (Fig. 5). In the presence of insulin, CtHtrA was also activated to 24-mers with little hexamer visible. The R299W mutant was present as hexamer and 24-mer in the control with no activator. Low levels of 24-mer were formed when R299W was incubated with **Act1** and mainly hexamer with insulin. The L302N mutant was not detected in any higher oligomeric form under any conditions. L302N was detected as faint hexamer and trimer but mostly monomer under all tested conditions (not visible on these gels). R373A incubation with **Act1** resulted in 24-mer formation. Conversely, R373A incubation with insulin was mainly detected as hexamer. I365D was detected as hexamers, trimers, 12-mers, and some 24-mers when incubated in the absence of activators. In contrast, no 12-mers, but only hexamers and 24-mers were detected when I365D was incubated with **Act1**.

The R373A/R299W protein was mainly detected as hexamers when not activated. **Act1** induced R373A/R299W 24-mer formation. This activation was very similar to that observed for CtHtrA, and was the only condition during which any of the mutated proteins oligomerised in a similar profile to CtHtrA. In direct contrast, R373A/R299W did not form a similar profile of oligomers as wild-type CtHtrA when incubated with insulin.

### Recombinant chlamydial major outer membrane protein (MOMP) and chlamydial outer membrane protein C-terminal peptides can induce CtHtrA oligomerisation

**Figure 6:**
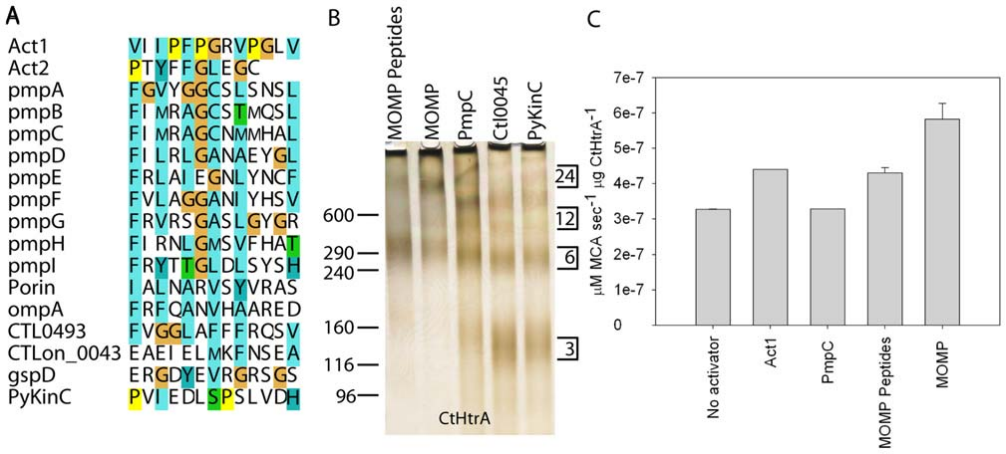
*Chlamydia trachomatis* major outer membrane protein (MOMP) induces CtHtrA oligomerisation and protease activation. A. Alignment of **Act1**, **Act2**, and PyKinC (control cytoplasmic enzyme) with C-terminal sequence of all outer membrane b-barrels in *Chlamydia trachomatis* L2 (note: ompA is MOMP). Residue numbers are indicated below the alignment with 1 being the most C-terminal residue. The alignment was performed in ClustalX with the default colour scheme hydrophobic: WLVIMAFC (blue), cysteine: C (pink), small: G (orange) cyclic: P (yellow), Polar hydrophilic: TSNQ (green), Polar Aromatic: HY (cyan), Acidic: ED (magenta), Basic: KR (red). B. Oligomerisation of CtHtrA after 10 mins incubation with MBP-MOMP, MBP-MOMP peptides, PmpC peptide, Ctl0043 peptide, and pyruvate kinase C peptide (PyKinC). C. CtHtrA proteolysis of **Bcas1** (rate y-axis) with each of the activators (indicated on x-axis).

The most likely *in vivo* candidates for the chaperone activity of CtHtrA are outer membrane ß-barrels, which have been previously identified for *E. coli* DegP [25]. The OMPdb outer membrane ß-barrel database [26] identifies 13 outer membrane ß-barrels in the *C. trachomatis* L2 genome. An alignment of these shows the expected high homology of the C-terminal sequences consisting of the recognition sequence for the outer membrane protein assembly apparatus BamA (reviewed [27]). Co-incidentally, the conserved hydrophobic residues for the BamA recognition motif are consistent with the hydrophobic residues in the **Act1** but not **Act2** (Fig. 6). This could imply that the C-terminal sequences which stimulate CtHtrA oligomerisation are the same as those required to direct the substrate to BamA to facilitate outer membrane protein assembly. In order to test this possibility two peptides corresponding to the C-terminal 13 residues of proteins with this ß-barrel C-terminal sequence were tested for activation of CtHtrA oligomerisation. The two peptides were from the outer membrane ß-barrels PmpC and CtL0043 (PmpC: H-LAHMMNCGARMIF-OH, CtL0043: H-AESNFKMLEIEAE-OH). Additionally a control peptide based on the C-terminal sequence of a soluble cytoplasmic enzyme pyruvate kinase was tested (PyKinC: H-HDVLSPSLDEINVP-OH). Purified recombinant *C. trachomatis* MOMP, the major outer membrane protein with a maltose binding protein tag (MBP-MOMP) [28] and a tryptic digest of the MBP-MOMP were also tested for CtHtrA activation.

When CtHtrA was incubated with the tryptic digested MBP-MOMP protein (MOMP peptides), much of the protein remained present as a hexamer with only a minor amount of 24-mer visible (Fig. 6). In contrast, when CtHtrA was incubated with the purified whole MBP-MOMP, CtHtrA was mainly present as a 24-mer with only a minor amount of hexamer visible. Thus the same protein present as tryptic peptides or as full length protein had a differential impact on triggering CtHtrA oligomerisation. The incubation of CtHtrA with the three different C-terminal peptides from *C. trachomatis* proteins all resulted in similar oligomer formation with no 24-mer detected. The PmpC peptide activated the formation of 12-mer. The CtL0043 and PyKinC peptides activated mainly trimer formation with some 12-mer present (Fig. 6). These data suggest that the specific sequence of the peptide is important for the binding and subsequent triggering of oligomerisation. Furthermore, it is not just the presence of the consensus for outer membrane protein assembly of the peptide that determines successful activation of CtHtrA oligomerisation.

Finally, in order to determine if the 24-mer (highest amount with MOMP protein, or Act1), or 12-mer, or both, corresponds to enhanced protease activity, the incubations of CtHtrA and these potential *in vivo* activators were used for protease assays with the **Bcas1** substrate (Fig. 6). Incubation with the PmpC peptide did not increase the rate of proteolysis of **Bcas1**. The incubation with Act1, MBP-MOMP peptides, or MBP-MOMP protein resulted in an increased proteolysis rate. The presence of the majority of CtHtrA as higher order oligomers correlated with an increased rate of proteolysis for CtHtrA, particularly when these oligomers were predominately 24-mers.

## Discussion

DegP is a remarkable protein which conducts proteolysis and chaperone activities critical for pathogenesis, outer membrane protein biogenesis, and stress survival for many bacteria. As the most conserved and best understood member of the HtrA protein family, the elucidation of DegP's activation via allosteric regulation and oligomeric formation of spheres and bowl like structure has also contributed significantly to general understanding of protein structure regulatory mechanisms [18], [19], [25], [29], [30]. Two DegP structural forms are thought to mediate chaperone activity; lipid associated bowls and oligomeric spheres [20], [30]. Very recently the lipid associated bowl form has been demonstrated to mediate chaperone activity for one substrate for *Bordetella* [31], providing evidence that these two structural models are relevant *in vivo*. However, in spite of these advances it remains unknown how DegP differentially targets substrates for degradation or chaperone activity. Our current study identified for the first time that formation of CtHtrA oligomers triggered by chaperone substrates requires the residues which form the interface between PDZ1 domain and the protease. Furthermore, we demonstrated that a potential *in vivo* chaperone substrate, a major pathogenesis protein (MOMP), triggered oligomer formation suggesting that CtHtrA has a critical role in outer membrane protein assembly.

Generally, the preferred sequences for CtHtrA hydrolysis were hydrophobic with non-polar residues found to be particularly preferred at P1. The specificity of the protease activity did not vary when a complex mix of peptides (PICS) or individual peptides were present. The residues which were most preferred (generally non-polar residues) were consistent with those likely to be only accessible during protein unfolding or protein stress conditions. CtHtrA had a preference for V/I/A at P1, which is somewhat similar to elastase-2 [32]. While less specific, CtHtrA also showed a preference for small hydrophobic residues (A/G/L) on the prime-side of the active site P1′, P3′–P4′.

CtHtrA proteolysis was able to be activated by the addition of a second peptide. The proteins with PDZ1 activator cleft and PDZ1:protease interface mutations were all defective for protease activation indicating the activation involved allosteric binding to PDZ1. This indicates that the PDZ1 activator cleft and PDZ1:protease interface function in the activation of CtHtrA proteolysis.

The activation of CtHtrA proteolysis was demonstrated to be via the formation of higher order oligomers, predominantly a 24-mer. The presence of some oligomeric CtHtrA, even in the absence of activator, may imply that protein contaminants were present in the purified protein. It is commonly reported that HtrA/DegP purifies with other proteins bound [25]. The CtHtrA mutation data in this study, especially the mutations in the PDZ1 cleft, demonstrate that I365N and L302D are clearly important in the oligomerisation process. The L302D was unable to assemble into oligomers under any of the conditions tested, while I365N exhibited reduced 24-mer formation compared with the wild-type protein. The reduced protease activity and partially conserved oligomerisation activity observed for these two mutations are consistent with the previously reported role of these residues for *E. coli* DegP [18]. R299W and R299W/R373A, which reside in the PDZ1:protease interface, oligomerised to 24-mers when incubated with the activator, similar the wild-type protein. Thus, the PDZ1 cleft is critical for oligomer formation of CtHtrA, which is consistent with the previous findings for DegP [18].

CtHtrA incubation with insulin, a previously demonstrated *in vitro* chaperone substrate of CtHtrA [3], resulted in 24-mer and higher molecular weight forms. These results indicate that oligomerisation activity is also important for chaperone substrates. However, the proteins with PDZ1 cleft or PDZ1:protease interface mutations did not oligomerise when incubated with insulin. The R299W/R373A mutant remained mainly as hexamer when incubated with insulin, unlike when it was incubated with the activator peptide and formed a 24-mer. This is in contrast to wild-type CtHtrA which formed detectable 24mers when incubated with either insulin or with **Act1**. These observations indicate that the PDZ1:protease interface is critical for the formation of oligomers when a protein (chaperone substrate) is the activator but does not impact on oligomer formation when the activator is a peptide. This may be simply due to the difference in size, i.e. insulin is two longer polypeptide chains, whereas Act1 is a 13-mer peptide. This is consistent with the previous finding that different sized activators triggered different sized oligomers for DegP (peptides activated 12-mer versus full length ß-casein activated 24-mer) [33]. However, since wild-type CtHtrA did form 24-mer in the presence of both peptide and protein there may be subtle differences in the activation mechanism from that reported for DegP. This implies that these residues or the role of these residues for the activated oligomers structural conformation, are important specifically for chaperone substrates for CtHtrA. This is the first time these residues have been identified to function for any DegP oligomer formation. This data may suggest that HtrA protein activation for polypeptide fragments (such as those which would be present during protein stress) could be different than the activation in response to full length proteins such as insulin. Similarly to the findings here, DegP has been demonstrated to be activated in the presence of two short peptide fragments (activator and substrate) or polypeptide fragments long enough to activate and be cleaved (such as those previously reported in the model termed the molecular ruler [25]). However, the differences observed for the PDZ:protease interface mutants activation when triggered with insulin is unique. This *in vitro* model may therefore providing clues on how this multi-tasking family of proteins distinguishes between proteolysis and chaperone substrates.

To provide clues to *in vivo* targets of CtHtrA, peptides containing the consensus sequence for outer membrane ß-barrel assembly were tested for activation of oligomerisation. *C. trachomatis* outer membrane protein (MBP-MOMP), tryptic peptides of the same protein, and peptides corresponding to C-terminal sequences of other *C. trachomatis* outer membrane proteins were all examined. The peptides from the outer membrane proteins, especially PmpC, were able to trigger 12-mer but not 24-mer formation. These 12-mers may still be a chaperone form and possibly these may have some relevance to the previously reported lipid bowl chaperone structure (Shen *et al.*, 2008). However, as we have previously experimentally validated that insulin is a CtHtrA chaperone substrate, and we demonstrate here that insulin triggers CtHtrA 24-mer, we can only confidently assign a chaperone function to the 24-mers. The full length MOMP protein (and not the peptides resulting from the tryptic digest of the same protein) was the most efficient trigger of 24-mer assembly and increased proteolysis (other than **Act1**). Thus MOMP, an important outer membrane ß-barrel protein for *Chlamydia*, is potentially an *in vivo* chaperone substrate of CtHtrA.

The combined findings from this study have proven that CtHtrA can be allosterically activated and that this activation involves formation of protein oligomers. The major finding from this study is that the PDZ1:protease interface and the PDZ1 cleft residues have distinct roles in the formation of higher order oligomers after binding of CtHtrA to chaperone or proteolysis substrates. This is the first evidence implying that these two regions (PDZ1 cleft or PDZ1:protease interface) may differentially facilitate or regulate the oligomer formation for any DegP or HtrA. Regardless of this potential regulatory function, this study has revealed novel residues important for the activation mechanism of *C. trachomatis* CtHtrA.

## Materials and Methods

### Bacterial cultures

*E. coli JM109* was used for all cloning and genetic manipulations. *E. coli* BL21 (DE3) was transformed with the protein expression plasmids and used for protein expression. *E. coli* was routinely cultured on LB broth or plates (1 L: 10 g NaCl, 5 g Yeast Extract, 10 g tryptone, pH 7.2) (1.5% w/v agar) with Ampicillin (where required) at 100 µg/ml.

### Site directed mutagenesis

The pETHtrA and pETS247A purified plasmid DNA was used as the template for the site directed mutagenesis PCRs as previously described [3]. The site directed mutagenesis PCRs were conducted using the QuickChange II site directed mutagenesis kit (Stratagene), in accordance with the provided instructions. A number of the site directed mutants were alternatively generated using Pfu DNA polymerase (Promega) as the PCR polymerase (5-phosphorylated primers) followed by *Dpn*1 digest of the template DNA for 2 h at 37°C, overnight incubation with T4 DNA Ligase (Promega), prior to transformation into JM109. The primers used for site directed mutagenesis are listed in Table S1. All plasmids were confirmed to contain the expected mutation with no other introduced errors in the coding sequence by sequencing prior to transformation into BL21 (DE3) for protein expression.

### Protein expression and purification

The protein expression and purification methods for CtHtrA have been previously reported [3]. Briefly, BL21 (DE3) pETHtrA cultures were grown at 30°C until an O.D. 600(nm) of 0.6 was reached. The protein expression was induced with 0.5 mM IPTG and the cultures were grown for a further 150 min prior to harvesting the cells. The cultures were then lysed by sonication, and the unlysed cells were removed by centrifugation at 4000× g for 12 min at 4°C. The His-tagged protein was then purified from the cellular lysates using Talon Resin (Clontech, Australia), as per the manufacturer's instructions. These conditions were used for the expression and purification of all site directed mutant proteins reported in this study. The purified proteins were examined by polyacrylamide gel electrophoresis to ensure homogeneity was observed and Coomassie staining prior to use in protease assays. The MBP-MOMP protein used in the oligomerisation assay was kindly supplied by Mr Connor O'Meara. The protein was recombinantly expressed in *E. coli* and purified using established protocols [28]. The protein has an N-terminal MBP sequence and *C. muridarum* MOMP, the C-terminal sequence of this MOMP (last 13 residues, is 100% conserved with *C. trachomatis* L2). MBP-MOMP was reduced, denatured, and digested to completion using trypsin. MOMP tryptic fragments were produced by reducing purified MOMP (1 mg/ml) in PBS with 5 mM DTT (Merck) for 1 h at 65°C followed by carboxyamidomethylation with 25 mM iodoacetamide for 30 min (SigmaAldrich, Australia) in the dark at room temperature. The reduced and alkylated protein was then digested with sequencing grade trypsin (Promega, Australia) for 18 h at 37°C at 1∶100 trypsin∶MOMP mass ratio. Digestion was stopped by heat deactivating trypsin for 2 h at 85°C and the reduction and carboxyamidomethylation repeated. The final peptide mixture was C18 purified using Waters SepPak (1 ml) columns according to manufacturer's instructions. Eluted peptides were vacuum concentrated to dryness in a Speedivac before reconstituting in MilliQ water for further use.

### Protease assays

The purified recombinant protein was examined by Coomassie stained polyacrylamide gels and activity was checked using ß-casein cleavage assays prior to use in peptide assays. Briefly, this involved incubating 2 µg of the protease with 10 µg of ß-casein in 50 mM Tris pH 7.0, 20 mM MgCl_2_ for 2 h prior to examining the assays by SDS PAGE (ß-casein and protease only controls were always conducted). Protease assays using peptides were conducted using custom synthesised peptides (Mimotopes). The peptides were purified to 95% purity (or greater) and were typically resuspended in 50% isopropanol, although some peptides required 100% DMSO. Protease assays using pNA labelled peptides were conducted using the xMARK Microplate spectrophotometer (Bio-Rad) at 405 nm, incubated at 37°C, with a reading taken every 10 s for 30 min. The maximum slope over a minimum of 8 readings was used for the data analysis. Other peptides were labelled with 7-methoxycoumarin-4-acetic acid (MCA, fluorophore) and 2,4-dinitrophenyl (DNP, quencher). The protease assays using these peptides were conducted in black plates at 37°C using the POLARstar Optima (BMG Labtech). The assays were excited at 340 nm and emission was detected at 405 nm with a gain of 1000, the assays were monitored every 20 s or at the minimum cycle time for up to 40 min. The Omega software was used to calculate the maximum rate from a minimum of 6 time points for each assay. The REPLi library of peptides (Mimotopes, Melbourne, Australia) was also assayed with CtHtrA in the POLARstar Optima using similar assay conditions to those described above. Substrate only and protease only assay controls were conducted for every experiment and protease assays were typically conducted in triplicate on at least two different days. The assay results shown are the maximal rate observed from three or more different assays and adjusted for protein concentration. The assay results shown for the fluorescent compounds have been normalised for the amount of fluorescent emission detected using a standard curve of known concentrations of the MCA fluorophore under the identical assay conditions. All of the peptides used in this work were synthesised commercially to 95% purity or greater (Mimotopes, Melbourne, Australia). All protease assays were conducted minimally in duplicate on three separate occasions.

### Oligomerisation Assays

The oligomerisation of HtrA into 12- and 24-mer complexes has been independently detected for *E. coli* HtrA (DegP). The oligomerisation of HtrA (DegP) was triggered by peptides from digested ß-casein [18]. Assays to detect whether CtHtrA and site directed mutant protein oligomerisation was triggered by substrates were conducted essentially as previously described (Merdanovic *et al.*, 2010). Briefly, 2 mg/ml protein solutions in 100 mM sodium phosphate buffer pH 7.0 were pre-incubated to the required temperature. The ligands (activator peptide, or insulin) were added and at 10 min, and the proteins were fixed using 0.5% glutaraldehyde. Samples were fixed at room temperature for 2 min prior to addition of 1 M Tris pH 7.5 to remove any excess glutaraldehyde. Samples were incubated for another 10 min at room temperature prior to addition of SDS PAGE reducing loading buffer. The protein samples were examined on 3–8% TrisAcetate gradient polyacrylamide gels (Novex, Invitrogen) by silver staining [34]. The proteins used in this study were not initially denatured by treatment with urea as, the refolding, formation of oligomers, and protease activity was never successfully detected with denatured CtHtrA. All assays were conducted in duplicate on three separate occasions with at least two gels of each experiment completed for each assay condition.

### Molecular Modelling of the CtHtrA structure

The crystal structures of activated *E. coli* DegS (pdb1soz and pdb2r3y) [35], [36] and the inactive *E. coli* DegP hexamer (pdb1ky9) [17] were used to generate homology models of CtHtrA (accession no. P18584). Models were generated using Modeller9v6 [37] based on sequence alignments generated using use T-coffee server [38].

### Proteomic identification of CtHtrA cleavage sites

The proteome-derived database searchable peptide library approach or Proteomic Identification of protease Cleavage Sites (PICS) was used to identify CtHtrA cleavage sites in a naturally sourced peptide library, as previously described by Schilling and Overall (2008) with some minor modifications. CtHtrA, 12 µM **Act1**, and peptide libraries were combined (100 mM HEPES pH 7.0 as the reaction buffer). All recovered peptides were analysed by LC/MS/MS at the Institute for Molecular Biosciences at the University of Queensland using a QStar Elite. All data was converted to mzXML using mzwiff and searched using Mascot and Tandem against the uniprot database (Jan 2010: Human and Mouse with included reverse decoy database). Data were searched using a precursor ion tolerance of +/−2.2 Da, MS/MS tolerance of 0.4 Da, fixed modifications including dimethylation of lysine, sulfamine modification of peptide N-termini, carboxyamidomethylation of cysteines and variable oxidation of methionines. All search results were analysed using the trans-proteomic pipeline (v4.4) combining both searches using the interprophet parser and corrected for false-discovery rate of less than or equal to 0.05. All raw data and mass spectra can be made available on request.

### MS and MS-MS analysis of peptide cleavage products

**Bcas1** and **Act1** peptides (150 µM concentration) digested with CtHtrA (71 µg) were analysed by online LC/MS/MS using a QStar Pulsar I (Applied Biosystems) in independent data acquisition mode. Data acquired were converted into mzXML format using proteowizard (v2) and searched using (v1, the global proteome machine, ftp://ftp.thegpm.org/fasta/cRAP), against CtHtrA and **Act1** and **Bcas1** peptide sequences with an included decoy database of the reverse sequence for each protein. The search also included potential mass modifications accounting for the additional quencher/fluorophore moieties on the N and C peptide termini, respectively. Matches with an expected ratio of <1 were considered identified. All raw data and mass spectra can be made available on request.

## Supporting Information

Figure S1 **A**. Weblog of the PICS results. **B**. A full listing of all PICs data (listed below).
(DOCX)

Figure S2 **Results of protease assays using the REPLI Library all wells which has detectable substrates for CtHtrA are shown on the graph.** The relative rate of fluorescent accumulation per min per ug of CtHtrA is shown on the Y axis. The well descriptions are indicated on the x axis. The wells contain 4–8 peptides with similar functional residues grouped, with a single residue variation between the two listed on each peptide. The peptides have a fluorophore (MCA) and quencher (Lys-DNP) with two trimers of glycine on each side of the variant trimer of amino acids in the middle (variant trimer ranges are indicated on the x axis).
(DOCX)

Figure S3 **Characterisation of Bcas1 cleavage by CtHtrA.** A. LC/MS plot of **Bcas1** alone. B. B LC/MS plot of **Bcas1** cleaved with CtHtrA shows addition of ions compared to **Bcas1** alone. C. MS/MS of product ion 552.37 m/z, 26.3 min (circle in B) matches internal fragment of **Bcas1**.
(DOCX)

Figure S4 **Characterisation of protease specificity in the presence of the activator peptide using PICS.** **A**. Web Logo summary of all peptide cleavage sites identified during PICS protocol with CtHtrA and **Act1**. **B**. The table below lists all peptide cleavage sites identified during PICS protocol with CtHtrA and **Act1**.
(DOCX)

Table S1 **Primers and amplification conditions used for site directed mutagenesis PCR.**
(DOCX)
