# Supplementary material for: Unique Residues Involved in Activation of the Multitasking Protease/Chaperone HtrA from Chlamydia trachomatis
Source: PLoS One. 2011 Sep 8;6(9):e24547. doi: 10.1371/journal.pone.0024547 (PMC3169616; doi:10.1371/journal.pone.0024547)
Supplement: Figure S1 — A. Weblog of the PICS results. B. A full listing of all PICs data (listed below). (DOCX) [file pone.0024547.s001.docx]

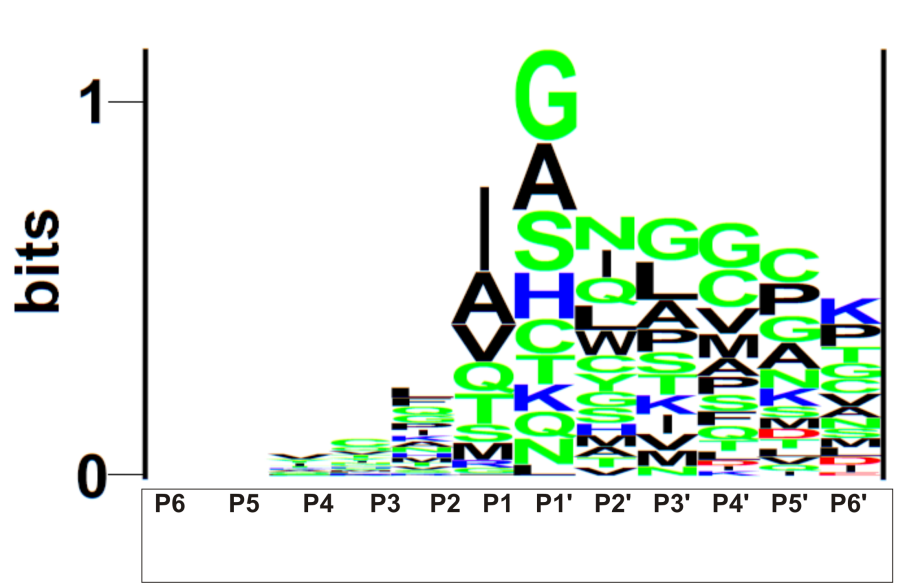


**Supporting information Fig S1A.**

**Supporting information Table S1B. Proteome wide identification of CtHtrA protease cleavage sites (PICS).**

| **probability** | **spectrum** | **probability** | **ions** | **peptide** | **protein** | **calc_neutral_pep_mass** | |
| --- | --- | --- | --- | --- | --- | --- | --- |
| 0.9568 | SS_20101013_QS_IDA_S_5_GluC.00753.00753.2 | 0.942515 | Nov-18 | S.n[89.01]C[160.03]GPPPELLNG.N | sp\|P08603\|CFAH_HUMAN | 1140.495 |  |
| 0.9199 | SS_20101013_QS_IDA_S_5_GluC.00925.00925.2 | 0.894756 | 15/22 | T.n[89.01]LSSSAK[156.13]ADTSK[156.13]P.S | sp\|Q9UHX3\|EMR2_HUMAN | 1334.675 |  |
| 0.9702 | SS_20101013_QS_IDA_S_5_GluC.00930.00930.2 | 0.960162 | 14/24 | R.n[89.01]ASASAAALAVFSE.V | sp\|Q8NCT1\|ARRD4_HUMAN | 1281.591 |  |
| 0.9961 | SS_20101013_QS_IDA_S_5_GluC.00986.00986.2 | 0.994739 | 22/24 | A.n[89.01]GLAGK[156.13]DPVQC[160.03]SRD.V | sp\|Q99497\|PARK7_HUMAN,sp\|Q99LX0\|PARK7_MOUSE | 1517.697 |  |
| 0.8985 | SS_20101013_QS_IDA_S_5_GluC.01018.01018.2 | 0.867605 | 17/30 | G.n[89.01]GIGGGGGGGGGGGGEE.E | sp\|Q8K1R7\|NEK9_MOUSE | 1218.457 |  |
| 0.9151 | SS_20101013_QS_IDA_S_5_GluC.01025.01025.2 | 0.888631 | 16/30 | D.n[89.01]ELGSGADASATQAARS.T | sp\|Q92673\|SORL_HUMAN | 1578.695 |  |
| 0.9956 | SS_20101013_QS_IDA_S_5_GluC.01072.01072.2 | 0.995882 | 23/30 | Q.n[89.01]ATVVAVGSGSK[156.13]GK[156.13]GGE.I | sp\|P61604\|CH10_HUMAN | 1546.803 |  |
| 0.9958 | SS_20101013_QS_IDA_S_5_GluC.01079.01079.2 | 0.996068 | 22/30 | Q.n[89.01]ATVVAVGSGSK[156.13]GK[156.13]GGE.I | sp\|P61604\|CH10_HUMAN | 1546.803 |  |
| 0.9378 | SS_20101013_QS_IDA_S_5_GluC.01146.01146.2 | 0.917772 | 16/20 | S.n[89.01]GVM[147.04]APPSGPTV.A | sp\|P22460\|KCNA5_HUMAN | 1115.499 |  |
| 0.98 | SS_20101013_QS_IDA_S_5_GluC.01238.01238.2 | 0.973172 | 18/18 | V.n[89.01]ANNQDK[156.13]LGFE.D | sp\|P09936\|UCHL1_HUMAN | 1250.56 |  |
| 0.8616 | SS_20101013_QS_IDA_S_5_GluC.01301.01301.2 | 0.821702 | 16/16 | D.n[89.01]K[156.13]SGSMGGK[156.13]D.R | sp\|Q14CN2\|CLCA4_HUMAN | 1009.458 |  |
| 0.9806 | SS_20101013_QS_IDA_S_5_GluC.01313.01313.2 | 0.973971 | 22/26 | A.n[89.01]SYAVQSK[156.13]YGDFNK[156.13]E.V | sp\|P26038\|MOES_HUMAN,sp\|P26041\|MOES_MOUSE | 1778.819 |  |
| 0.8951 | SS_20101013_QS_IDA_S_5_GluC.01395.01395.2 | 0.863327 | 15/14 | I.n[89.01]SHLVIMHE.E | sp\|P18206\|VINC_HUMAN,sp\|Q64727\|VINC_MOUSE | 1052.479 |  |
| 0.9315 | SS_20101013_QS_IDA_S_5_GluC.01403.01403.2 | 0.996195 | 23/26 | A.n[89.01]STGAAK[156.13]AVGK[156.13]VIPE.L | sp\|P04406\|G3P_HUMAN,sp\|Q64467\|G3PT_MOUSE,sp\|P16858\|G3P_MOUSE | 1470.812 |  |
| 0.9779 | SS_20101013_QS_IDA_S_5_GluC.01411.01411.2 | 0.970376 | 21/20 | V.n[89.01]SLAVC[160.03]K[156.13]AGAVE.K | sp\|P06733\|ENOA_HUMAN,sp\|P17182\|ENOA_MOUSE | 1219.594 |  |
| 0.9727 | SS_20101013_QS_IDA_S_5_GluC.01412.01412.2 | 0.963472 | 18/18 | A.n[89.01]GIIASARAGE.T | sp\|P13639\|EF2_HUMAN,sp\|P58252\|EF2_MOUSE | 1031.507 |  |
| 0.9986 | SS_20101013_QS_IDA_S_5_GluC.01419.01419.2 | 0.999904 | Aug-26 | S.n[89.01]STGAAK[156.13]AVGK[156.13]VIPE.L | sp\|Q64467\|G3PT_MOUSE,sp\|P04406\|G3P_HUMAN,sp\|P16858\|G3P_MOUSE | 1470.819 |  |
| 0.1681 | SS_20101013_QS_IDA_S_5_GluC.01427.01427.2 | 0.985914 | Jun-26 | S.n[89.01]STGAAK[156.13]AVGK[156.13]VIPE.L | sp\|Q64467\|G3PT_MOUSE,sp\|P04406\|G3P_HUMAN,sp\|P16858\|G3P_MOUSE | 1470.819 |  |
| 0.0946 | SS_20101013_QS_IDA_S_5_GluC.01435.01435.2 | 0.975887 | May-26 | S.n[89.01]STGAAK[156.13]AVGK[156.13]VIPE.L | sp\|Q64467\|G3PT_MOUSE,sp\|P04406\|G3P_HUMAN,sp\|P16858\|G3P_MOUSE | 1470.819 |  |
| 0.9673 | SS_20101013_QS_IDA_S_5_GluC.01443.01443.2 | 0.997963 | Jun-26 | S.n[89.01]STGAAK[156.13]AVGK[156.13]VIPE.L | sp\|Q64467\|G3PT_MOUSE,sp\|P04406\|G3P_HUMAN,sp\|P16858\|G3P_MOUSE | 1470.819 |  |
| 0.9965 | SS_20101013_QS_IDA_S_5_GluC.01451.01451.2 | 0.999761 | Aug-26 | S.n[89.01]STGAAK[156.13]AVGK[156.13]VIPE.L | sp\|Q64467\|G3PT_MOUSE,sp\|P04406\|G3P_HUMAN,sp\|P16858\|G3P_MOUSE | 1470.819 |  |
| 0.9842 | SS_20101013_QS_IDA_S_5_GluC.01460.01460.2 | 0.999016 | 24/26 | A.n[89.01]STGAAK[156.13]AVGK[156.13]VIPE.L | sp\|P04406\|G3P_HUMAN,sp\|Q64467\|G3PT_MOUSE,sp\|P16858\|G3P_MOUSE | 1470.812 |  |
| 0.9339 | SS_20101013_QS_IDA_S_5_GluC.01475.01475.2 | 0.9963 | 25/26 | A.n[89.01]STGAAK[156.13]AVGK[156.13]VIPE.L | sp\|P04406\|G3P_HUMAN,sp\|Q64467\|G3PT_MOUSE,sp\|P16858\|G3P_MOUSE | 1470.812 |  |
| 0.92 | SS_20101013_QS_IDA_S_5_GluC.01506.01506.2 | 0.920883 | 20/22 | A.n[89.01]QATGK[156.13]PPQYIAV.H | sp\|P14174\|MIF_HUMAN | 1387.717 |  |
| 0.9416 | SS_20101013_QS_IDA_S_5_GluC.01514.01514.2 | 0.941134 | 22/22 | A.n[89.01]QATGK[156.13]PPQYIAV.H | sp\|P14174\|MIF_HUMAN | 1387.717 |  |
| 0.994 | SS_20101013_QS_IDA_S_5_GluC.01561.01561.2 | 0.994934 | 18/16 | Q.n[89.01]AVLLPK[156.13]K[156.13]TE.S | sp\|Q96QV6\|H2A1A_HUMAN,sp\|P04908\|H2A1B_HUMAN,sp\|Q93077\|H2A1C_HUMAN,sp\|P20671\|H2A1D_HUMAN,sp\|Q96KK5\|H2A1H_HUMAN,sp\|Q99878\|H2A1J_HUMAN,sp\|P0C0S8\|H2A1_HUMAN,sp\|Q6FI13\|H2A2A_HUMAN,sp\|Q8IUE6\|H2A2B_HUMAN,sp\|Q16777\|H2A2C_HUMAN,sp\|Q7L7L0\|H2A3_HUMAN,sp\|Q9BTM1\|H2AJ_HUMAN,sp\|Q8CGP5\|H2A1F_MOUSE,sp\|Q8CGP6\|H2A1H_MOUSE,sp\|Q8CGP7\|H2A1K_MOUSE,sp\|P22752\|H2A1_MOUSE,sp\|Q6GSS7\|H2A2A_MOUSE,sp\|Q64522\|H2A2B_MOUSE,sp\|Q64523\|H2A2C_MOUSE,sp\|Q8BFU2\|H2A3_MOUSE,sp\|Q8R1M2\|H2AJ_MOUSE | 1141.678 |  |
| 0.8766 | SS_20101013_QS_IDA_S_5_GluC.01569.01569.2 | 0.883417 | May-16 | Q.n[89.01]AVLLPK[156.13]K[156.13]TE.S | sp\|Q96QV6\|H2A1A_HUMAN,sp\|P04908\|H2A1B_HUMAN,sp\|Q93077\|H2A1C_HUMAN,sp\|P20671\|H2A1D_HUMAN,sp\|Q96KK5\|H2A1H_HUMAN,sp\|Q99878\|H2A1J_HUMAN,sp\|P0C0S8\|H2A1_HUMAN,sp\|Q6FI13\|H2A2A_HUMAN,sp\|Q8IUE6\|H2A2B_HUMAN,sp\|Q16777\|H2A2C_HUMAN,sp\|Q7L7L0\|H2A3_HUMAN,sp\|Q9BTM1\|H2AJ_HUMAN,sp\|Q8CGP5\|H2A1F_MOUSE,sp\|Q8CGP6\|H2A1H_MOUSE,sp\|Q8CGP7\|H2A1K_MOUSE,sp\|P22752\|H2A1_MOUSE,sp\|Q6GSS7\|H2A2A_MOUSE,sp\|Q64522\|H2A2B_MOUSE,sp\|Q64523\|H2A2C_MOUSE,sp\|Q8BFU2\|H2A3_MOUSE,sp\|Q8R1M2\|H2AJ_MOUSE | 1141.686 |  |
| 0.9937 | SS_20101013_QS_IDA_S_5_GluC.01570.01570.2 | 0.991508 | 20/22 | I.n[89.01]GGIGTVPVGRVE.T | sp\|P68104\|EF1A1_HUMAN,sp\|Q05639\|EF1A2_HUMAN,sp\|Q5VTE0\|EF1A3_HUMAN,sp\|P10126\|EF1A1_MOUSE,sp\|P62631\|EF1A2_MOUSE | 1227.628 |  |
| 0.8834 | SS_20101013_QS_IDA_S_5_GluC.01613.01613.2 | 0.848682 | 21/22 | I.n[89.01]GLGFK[156.13]TPK[156.13]EAIE.G | sp\|P62280\|RS11_HUMAN,sp\|P62281\|RS11_MOUSE | 1432.764 |  |
| 0.9607 | SS_20101013_QS_IDA_S_5_GluC.01638.01638.2 | 0.947634 | 20/28 | E.n[89.01]GSIK[156.13]GAGAISNFLPN.N | sp\|Q9UHC9\|NPCL1_HUMAN | 1560.797 |  |
| 0.9612 | SS_20101013_QS_IDA_S_5_GluC.01681.01681.2 | 0.963333 | 19/18 | I.n[89.01]K[156.13]LGLGIDEDE.V | sp\|P08238\|HS90B_HUMAN,sp\|P11499\|HS90B_MOUSE | 1203.57 |  |
| 0.9106 | SS_20101013_QS_IDA_S_5_GluC.01683.01683.2 | 0.882908 | 18/26 | S.n[89.01]SSSASSSPSSLGPE.L | sp\|Q5VZ18\|SHE_HUMAN,sp\|Q8BSD5\|SHE_MOUSE | 1366.556 |  |
| 0.9884 | SS_20101013_QS_IDA_S_5_GluC.01687.01687.2 | 0.988753 | 19/18 | I.n[89.01]K[156.13]LGLGIDEDE.V | sp\|P08238\|HS90B_HUMAN,sp\|P11499\|HS90B_MOUSE | 1203.57 |  |
| 0.9655 | SS_20101013_QS_IDA_S_5_GluC.01766.01766.2 | 0.953953 | 23/34 | V.n[89.01]LLGPPGAGK[156.13]GTQAPRLAE.N | sp\|P54819\|KAD2_HUMAN | 1847.993 |  |
| 0.8595 | SS_20101013_QS_IDA_S_5_GluC.01783.01783.2 | 0.819123 | 24/26 | E.n[89.01]VGDGTTSVVIIAAE.L | sp\|P17987\|TCPA_HUMAN,sp\|P11984\|TCPA1_MOUSE,sp\|P11983\|TCPA2_MOUSE | 1418.697 |  |
| 0.9862 | SS_20101013_QS_IDA_S_5_GluC.01799.01799.2 | 0.987051 | 21/24 | I.n[89.01]GIPPAPRGVPQIE.V | sp\|P38646\|GRP75_HUMAN,sp\|P11021\|GRP78_HUMAN,sp\|P34931\|HS71L_HUMAN,sp\|P08107\|HSP71_HUMAN,sp\|P54652\|HSP72_HUMAN,sp\|P17066\|HSP76_HUMAN,sp\|P11142\|HSP7C_HUMAN,sp\|P38647\|GRP75_MOUSE,sp\|P20029\|GRP78_MOUSE,sp\|Q61696\|HS71A_MOUSE,sp\|P17879\|HS71B_MOUSE,sp\|P16627\|HS71L_MOUSE,sp\|P17156\|HSP72_MOUSE,sp\|P63017\|HSP7C_MOUSE | 1417.739 |  |
| 0.9939 | SS_20101013_QS_IDA_S_5_GluC.01808.01808.2 | 0.994233 | 22/24 | I.n[89.01]GIPPAPRGVPQIE.V | sp\|P38646\|GRP75_HUMAN,sp\|P11021\|GRP78_HUMAN,sp\|P34931\|HS71L_HUMAN,sp\|P08107\|HSP71_HUMAN,sp\|P54652\|HSP72_HUMAN,sp\|P17066\|HSP76_HUMAN,sp\|P11142\|HSP7C_HUMAN,sp\|P38647\|GRP75_MOUSE,sp\|P20029\|GRP78_MOUSE,sp\|Q61696\|HS71A_MOUSE,sp\|P17879\|HS71B_MOUSE,sp\|P16627\|HS71L_MOUSE,sp\|P17156\|HSP72_MOUSE,sp\|P63017\|HSP7C_MOUSE | 1417.739 |  |
| 0.9968 | SS_20101013_QS_IDA_S_5_GluC.01826.01826.2 | 0.997007 | 23/24 | T.n[89.01]GYPGDK[156.13]PVATMWE.S | sp\|SSPA_STAAU\| | 1565.69 |  |
| 0.9186 | SS_20101013_QS_IDA_S_5_GluC.01831.01831.2 | 0.951733 | 30/32 | M.n[89.01]K[156.13]QTIGNSC[160.03]GTIGLIHAV.A | sp\|P09936\|UCHL1_HUMAN,sp\|Q9R0P9\|UCHL1_MOUSE | 1883.96 |  |
| 0.9963 | SS_20101013_QS_IDA_S_5_GluC.01834.01834.2 | 0.996541 | 20/24 | T.n[89.01]GYPGDK[156.13]PVATMWE.S | sp\|SSPA_STAAU\| | 1565.69 |  |
| 0.9527 | SS_20101013_QS_IDA_S_5_GluC.01838.01838.2 | 0.970768 | 30/32 | M.n[89.01]K[156.13]QTIGNSC[160.03]GTIGLIHAV.A | sp\|P09936\|UCHL1_HUMAN,sp\|Q9R0P9\|UCHL1_MOUSE | 1883.96 |  |
| 0.9005 | SS_20101013_QS_IDA_S_5_GluC.01844.01844.2 | 0.942312 | 24/32 | M.n[89.01]K[156.13]QTIGNSC[160.03]GTIGLIHAV.A | sp\|P09936\|UCHL1_HUMAN,sp\|Q9R0P9\|UCHL1_MOUSE | 1883.96 |  |
| 0.9593 | SS_20101013_QS_IDA_S_5_GluC.01852.01852.2 | 0.945795 | 27/30 | I.n[89.01]NNLGTIAK[156.13]SGTK[156.13]AFME.A | sp\|Q14568\|HS902_HUMAN,sp\|P07900\|HS90A_HUMAN,sp\|P08238\|HS90B_HUMAN,sp\|P07901\|HS90A_MOUSE,sp\|P11499\|HS90B_MOUSE | 1824.912 |  |
| 0.964 | SS_20101013_QS_IDA_S_5_GluC.01857.01857.2 | 0.965005 | 22/26 | M.n[89.01]K[156.13]QTIGNSC[160.03]GTIGLI.H | sp\|P09936\|UCHL1_HUMAN,sp\|Q9R0P9\|UCHL1_MOUSE | 1576.796 |  |
| 0.9593 | SS_20101013_QS_IDA_S_5_GluC.01864.01864.2 | 0.960608 | 24/26 | M.n[89.01]K[156.13]QTIGNSC[160.03]GTIGLI.H | sp\|P09936\|UCHL1_HUMAN,sp\|Q9R0P9\|UCHL1_MOUSE | 1576.796 |  |
| 0.9901 | SS_20101013_QS_IDA_S_5_GluC.01868.01868.2 | 0.986673 | 20/24 | A.n[89.01]AILMGDK[156.13]SENVQD.L | sp\|P08107\|HSP71_HUMAN,sp\|Q61696\|HS71A_MOUSE,sp\|P17879\|HS71B_MOUSE | 1534.701 |  |
| 0.9242 | SS_20101013_QS_IDA_S_5_GluC.01886.01886.2 | 0.927632 | 21/24 | I.n[89.01]GNSC[160.03]GTIGLIHAV.A | sp\|P09936\|UCHL1_HUMAN,sp\|Q9R0P9\|UCHL1_MOUSE | 1385.643 |  |
| 0.9799 | SS_20101013_QS_IDA_S_5_GluC.01893.01893.2 | 0.979825 | 23/24 | I.n[89.01]GNSC[160.03]GTIGLIHAV.A | sp\|P09936\|UCHL1_HUMAN,sp\|Q9R0P9\|UCHL1_MOUSE | 1385.643 |  |
| 0.9814 | SS_20101013_QS_IDA_S_5_GluC.01932.01932.2 | 0.975037 | 20/28 | E.n[89.01]RGIDGLRGPPGPQGD.P | sp\|Q63870\|CO7A1_MOUSE | 1578.758 |  |
| 0.9409 | SS_20101013_QS_IDA_S_5_GluC.01933.01933.2 | 0.921787 | 21/18 | I.n[89.01]SILGK[156.13]SLADE.L | sp\|P07195\|LDHB_HUMAN,sp\|P16125\|LDHB_MOUSE | 1147.58 |  |
| 0.9894 | SS_20101013_QS_IDA_S_5_GluC.01996.01996.2 | 0.985734 | 24/26 | Q.n[89.01]AAILMGDK[156.13]SENVQD.L | sp\|P08107\|HSP71_HUMAN,sp\|Q61696\|HS71A_MOUSE,sp\|P17879\|HS71B_MOUSE | 1605.738 |  |
| 0.9937 | SS_20101013_QS_IDA_S_5_GluC.02069.02069.2 | 0.991508 | 23/30 | I.n[89.01]TLK[156.13]TPLVSSPMDTVTE.A | sp\|P12268\|IMDH2_HUMAN,sp\|P24547\|IMDH2_MOUSE | 1833.911 |  |
| 0.9798 | SS_20101013_QS_IDA_S_5_GluC.02113.02113.2 | 0.980648 | 24/22 | I.n[89.01]AQGGVLPNIQAV.L | sp\|Q96QV6\|H2A1A_HUMAN,sp\|P04908\|H2A1B_HUMAN,sp\|Q93077\|H2A1C_HUMAN,sp\|P20671\|H2A1D_HUMAN,sp\|Q96KK5\|H2A1H_HUMAN,sp\|Q99878\|H2A1J_HUMAN,sp\|P0C0S8\|H2A1_HUMAN,sp\|Q6FI13\|H2A2A_HUMAN,sp\|Q8IUE6\|H2A2B_HUMAN,sp\|Q16777\|H2A2C_HUMAN,sp\|Q7L7L0\|H2A3_HUMAN,sp\|Q9BTM1\|H2AJ_HUMAN,sp\|P16104\|H2AX_HUMAN,sp\|Q8CGP5\|H2A1F_MOUSE,sp\|Q8CGP6\|H2A1H_MOUSE,sp\|Q8CGP7\|H2A1K_MOUSE,sp\|P22752\|H2A1_MOUSE,sp\|Q6GSS7\|H2A2A_MOUSE,sp\|Q64522\|H2A2B_MOUSE,sp\|Q64523\|H2A2C_MOUSE,sp\|Q8BFU2\|H2A3_MOUSE,sp\|Q8R1M2\|H2AJ_MOUSE,sp\|P27661\|H2AX_MOUSE | 1253.644 |  |
| 0.9737 | SS_20101013_QS_IDA_S_5_GluC.02120.02120.2 | 0.974949 | 23/22 | I.n[89.01]AQGGVLPNIQAV.L | sp\|Q96QV6\|H2A1A_HUMAN,sp\|P04908\|H2A1B_HUMAN,sp\|Q93077\|H2A1C_HUMAN,sp\|P20671\|H2A1D_HUMAN,sp\|Q96KK5\|H2A1H_HUMAN,sp\|Q99878\|H2A1J_HUMAN,sp\|P0C0S8\|H2A1_HUMAN,sp\|Q6FI13\|H2A2A_HUMAN,sp\|Q8IUE6\|H2A2B_HUMAN,sp\|Q16777\|H2A2C_HUMAN,sp\|Q7L7L0\|H2A3_HUMAN,sp\|Q9BTM1\|H2AJ_HUMAN,sp\|P16104\|H2AX_HUMAN,sp\|Q8CGP5\|H2A1F_MOUSE,sp\|Q8CGP6\|H2A1H_MOUSE,sp\|Q8CGP7\|H2A1K_MOUSE,sp\|P22752\|H2A1_MOUSE,sp\|Q6GSS7\|H2A2A_MOUSE,sp\|Q64522\|H2A2B_MOUSE,sp\|Q64523\|H2A2C_MOUSE,sp\|Q8BFU2\|H2A3_MOUSE,sp\|Q8R1M2\|H2AJ_MOUSE,sp\|P27661\|H2AX_MOUSE | 1253.644 |  |
| 0.8419 | SS_20101013_QS_IDA_S_5_GluC.02121.02121.2 | 0.829635 | 24/26 | V.n[89.01]ANLFNK[156.13]YPALTK[156.13]PE.N | sp\|P13797\|PLST_HUMAN,sp\|Q99K51\|PLST_MOUSE | 1748.917 |  |
| 0.8272 | SS_20101013_QS_IDA_S_5_GluC.02128.02128.2 | 0.81587 | 24/26 | V.n[89.01]ANLFNK[156.13]YPALTK[156.13]PE.N | sp\|P13797\|PLST_HUMAN,sp\|Q99K51\|PLST_MOUSE | 1748.917 |  |
| 0.9859 | SS_20101013_QS_IDA_S_5_GluC.02165.02165.2 | 0.981079 | 21/26 | I.n[89.01]AAIC[160.03]AGPTALLAHE.I | sp\|Q99497\|PARK7_HUMAN,sp\|Q99LX0\|PARK7_MOUSE | 1481.701 |  |
| 0.9516 | SS_20101013_QS_IDA_S_5_GluC.02201.02201.2 | 0.935711 | 20/18 | A.n[89.01]GQSVLLQLPQ.- | sp\|P35232\|PHB_HUMAN,sp\|P67778\|PHB_MOUSE | 1169.612 |  |
| 0.9609 | SS_20101013_QS_IDA_S_5_GluC.02231.02231.2 | 0.947897 | 26/32 | V.n[89.01]SNASC[160.03]TTNC[160.03]LAPLAK[156.13]VI.H | sp\|O14556\|G3PT_HUMAN,sp\|P04406\|G3P_HUMAN,sp\|Q64467\|G3PT_MOUSE,sp\|P16858\|G3P_MOUSE | 1934.927 |  |
| 0.9773 | SS_20101013_QS_IDA_S_5_GluC.02279.02279.2 | 0.972649 | 24/26 | A.n[89.01]SC[160.03]TTNC[160.03]LAPLAK[156.13]VI.H | sp\|O14556\|G3PT_HUMAN,sp\|P04406\|G3P_HUMAN,sp\|Q64467\|G3PT_MOUSE,sp\|P16858\|G3P_MOUSE | 1662.815 |  |
| 0.9862 | SS_20101013_QS_IDA_S_5_GluC.02327.02327.2 | 0.986467 | 26/30 | A.n[89.01]TLK[156.13]MSVQPTVSLGGFE.I | sp\|P06748\|NPM_HUMAN,sp\|Q61937\|NPM_MOUSE | 1808.906 |  |
| 0.9781 | SS_20101013_QS_IDA_S_5_GluC.02329.02329.2 | 0.970318 | Aug-26 | A.n[89.01]ALK[156.13]K[156.13]ALAAAGYDVE.K | sp\|Q02539\|H11_HUMAN,sp\|P16403\|H12_HUMAN,sp\|P16402\|H13_HUMAN,sp\|P10412\|H14_HUMAN,sp\|P22492\|H1T_HUMAN,sp\|P15864\|H12_MOUSE,sp\|P43277\|H13_MOUSE,sp\|P43274\|H14_MOUSE,sp\|Q07133\|H1T_MOUSE | 1562.846 |  |
| 0.8125 | SS_20101013_QS_IDA_S_5_GluC.02335.02335.2 | 0.821461 | 24/30 | A.n[89.01]TLK[156.13]MSVQPTVSLGGFE.I | sp\|P06748\|NPM_HUMAN,sp\|Q61937\|NPM_MOUSE | 1808.906 |  |
| 0.7523 | SS_20101013_QS_IDA_S_5_GluC.02425.02425.2 | 0.808459 | 35/36 | T.n[89.01]HSLGGGTGSGMGTLLISK[156.13]I.R | sp\|Q13885\|TBB2A_HUMAN,sp\|Q9BVA1\|TBB2B_HUMAN,sp\|P68371\|TBB2C_HUMAN,sp\|P04350\|TBB4_HUMAN,sp\|P07437\|TBB5_HUMAN,sp\|Q9BUF5\|TBB6_HUMAN,sp\|A6NNZ2\|TBB8B_HUMAN,sp\|Q7TMM9\|TBB2A_MOUSE,sp\|Q9CWF2\|TBB2B_MOUSE,sp\|P68372\|TBB2C_MOUSE,sp\|Q9D6F9\|TBB4_MOUSE,sp\|P99024\|TBB5_MOUSE,sp\|Q922F4\|TBB6_MOUSE | 1900.975 |  |
| 0.7874 | SS_20101013_QS_IDA_S_5_GluC.02433.02433.2 | 0.830632 | 36/36 | T.n[89.01]HSLGGGTGSGMGTLLISK[156.13]I.R | sp\|Q13885\|TBB2A_HUMAN,sp\|Q9BVA1\|TBB2B_HUMAN,sp\|P68371\|TBB2C_HUMAN,sp\|P04350\|TBB4_HUMAN,sp\|P07437\|TBB5_HUMAN,sp\|Q9BUF5\|TBB6_HUMAN,sp\|A6NNZ2\|TBB8B_HUMAN,sp\|Q7TMM9\|TBB2A_MOUSE,sp\|Q9CWF2\|TBB2B_MOUSE,sp\|P68372\|TBB2C_MOUSE,sp\|Q9D6F9\|TBB4_MOUSE,sp\|P99024\|TBB5_MOUSE,sp\|Q922F4\|TBB6_MOUSE | 1900.975 |  |
| 0.8345 | SS_20101013_QS_IDA_S_5_GluC.02450.02450.2 | 0.848377 | 26/28 | I.n[89.01]HWGGVPNEFNGAVFI.N | sp\|SSPA_STAAU\| | 1730.788 |  |
| 0.7672 | SS_20101013_QS_IDA_S_5_GluC.02458.02458.2 | 0.798099 | 24/28 | I.n[89.01]HWGGVPNEFNGAVFI.N | sp\|SSPA_STAAU\| | 1730.788 |  |
| 0.9846 | SS_20101013_QS_IDA_S_5_GluC.02597.02597.3 | 0.979309 | May-68 | A.n[89.01]QGGVLPNIQAVLLPK[156.13]K[156.13]TE.S | sp\|Q96QV6\|H2A1A_HUMAN,sp\|P04908\|H2A1B_HUMAN,sp\|Q93077\|H2A1C_HUMAN,sp\|P20671\|H2A1D_HUMAN,sp\|Q96KK5\|H2A1H_HUMAN,sp\|Q99878\|H2A1J_HUMAN,sp\|P0C0S8\|H2A1_HUMAN,sp\|Q6FI13\|H2A2A_HUMAN,sp\|Q8IUE6\|H2A2B_HUMAN,sp\|Q16777\|H2A2C_HUMAN,sp\|Q7L7L0\|H2A3_HUMAN,sp\|Q9BTM1\|H2AJ_HUMAN,sp\|Q8CGP5\|H2A1F_MOUSE,sp\|Q8CGP6\|H2A1H_MOUSE,sp\|Q8CGP7\|H2A1K_MOUSE,sp\|P22752\|H2A1_MOUSE,sp\|Q6GSS7\|H2A2A_MOUSE,sp\|Q64522\|H2A2B_MOUSE,sp\|Q64523\|H2A2C_MOUSE,sp\|Q8BFU2\|H2A3_MOUSE,sp\|Q8R1M2\|H2AJ_MOUSE | 2048.178 |  |
| 0.9139 | SS_20101013_QS_IDA_S_5_GluC.02762.02762.2 | 0.887103 | 27/28 | A.n[89.01]K[156.13]ALANVNIGSLIC[160.03]NV.G | sp\|P05386\|RLA1_HUMAN,sp\|P47955\|RLA1_MOUSE | 1700.896 |  |
| 0.9842 | SS_20101013_QS_IDA_S_5_GluC.02790.02790.2 | 0.979001 | 30/32 | Q.n[89.01]GGVLPNIQAVLLPK[156.13]K[156.13]TE.S | sp\|Q96QV6\|H2A1A_HUMAN,sp\|P04908\|H2A1B_HUMAN,sp\|Q93077\|H2A1C_HUMAN,sp\|P20671\|H2A1D_HUMAN,sp\|Q96KK5\|H2A1H_HUMAN,sp\|Q99878\|H2A1J_HUMAN,sp\|P0C0S8\|H2A1_HUMAN,sp\|Q6FI13\|H2A2A_HUMAN,sp\|Q8IUE6\|H2A2B_HUMAN,sp\|Q16777\|H2A2C_HUMAN,sp\|Q7L7L0\|H2A3_HUMAN,sp\|Q9BTM1\|H2AJ_HUMAN,sp\|Q8CGP5\|H2A1F_MOUSE,sp\|Q8CGP6\|H2A1H_MOUSE,sp\|Q8CGP7\|H2A1K_MOUSE,sp\|P22752\|H2A1_MOUSE,sp\|Q6GSS7\|H2A2A_MOUSE,sp\|Q64522\|H2A2B_MOUSE,sp\|Q64523\|H2A2C_MOUSE,sp\|Q8BFU2\|H2A3_MOUSE,sp\|Q8R1M2\|H2AJ_MOUSE | 1920.112 |  |
| 0.9136 | SS_20101013_QS_IDA_S_5_GluC.02798.02798.2 | 0.891607 | 26/32 | Q.n[89.01]GGVLPNIQAVLLPK[156.13]K[156.13]TE.S | sp\|Q96QV6\|H2A1A_HUMAN,sp\|P04908\|H2A1B_HUMAN,sp\|Q93077\|H2A1C_HUMAN,sp\|P20671\|H2A1D_HUMAN,sp\|Q96KK5\|H2A1H_HUMAN,sp\|Q99878\|H2A1J_HUMAN,sp\|P0C0S8\|H2A1_HUMAN,sp\|Q6FI13\|H2A2A_HUMAN,sp\|Q8IUE6\|H2A2B_HUMAN,sp\|Q16777\|H2A2C_HUMAN,sp\|Q7L7L0\|H2A3_HUMAN,sp\|Q9BTM1\|H2AJ_HUMAN,sp\|Q8CGP5\|H2A1F_MOUSE,sp\|Q8CGP6\|H2A1H_MOUSE,sp\|Q8CGP7\|H2A1K_MOUSE,sp\|P22752\|H2A1_MOUSE,sp\|Q6GSS7\|H2A2A_MOUSE,sp\|Q64522\|H2A2B_MOUSE,sp\|Q64523\|H2A2C_MOUSE,sp\|Q8BFU2\|H2A3_MOUSE,sp\|Q8R1M2\|H2AJ_MOUSE | 1920.112 |  |
| 0.9925 | SS_20101013_QS_IDA_S_5_GluC.02820.02820.3 | 0.998442 | Jul-80 | V.n[89.01]TIAQGGVLPNIQAVLLPK[156.13]K[156.13]TE.S | sp\|Q96QV6\|H2A1A_HUMAN,sp\|P04908\|H2A1B_HUMAN,sp\|Q93077\|H2A1C_HUMAN,sp\|P20671\|H2A1D_HUMAN,sp\|Q96KK5\|H2A1H_HUMAN,sp\|Q99878\|H2A1J_HUMAN,sp\|P0C0S8\|H2A1_HUMAN,sp\|Q6FI13\|H2A2A_HUMAN,sp\|Q8IUE6\|H2A2B_HUMAN,sp\|Q16777\|H2A2C_HUMAN,sp\|Q7L7L0\|H2A3_HUMAN,sp\|Q9BTM1\|H2AJ_HUMAN,sp\|Q8CGP5\|H2A1F_MOUSE,sp\|Q8CGP6\|H2A1H_MOUSE,sp\|Q8CGP7\|H2A1K_MOUSE,sp\|P22752\|H2A1_MOUSE,sp\|Q6GSS7\|H2A2A_MOUSE,sp\|Q64522\|H2A2B_MOUSE,sp\|Q64523\|H2A2C_MOUSE,sp\|Q8BFU2\|H2A3_MOUSE,sp\|Q8R1M2\|H2AJ_MOUSE | 2333.347 |  |
| 0.8828 | SS_20101013_QS_IDA_S_5_GluC.02826.02826.3 | 0.98135 | Jul-80 | V.n[89.01]TIAQGGVLPNIQAVLLPK[156.13]K[156.13]TE.S | sp\|Q96QV6\|H2A1A_HUMAN,sp\|P04908\|H2A1B_HUMAN,sp\|Q93077\|H2A1C_HUMAN,sp\|P20671\|H2A1D_HUMAN,sp\|Q96KK5\|H2A1H_HUMAN,sp\|Q99878\|H2A1J_HUMAN,sp\|P0C0S8\|H2A1_HUMAN,sp\|Q6FI13\|H2A2A_HUMAN,sp\|Q8IUE6\|H2A2B_HUMAN,sp\|Q16777\|H2A2C_HUMAN,sp\|Q7L7L0\|H2A3_HUMAN,sp\|Q9BTM1\|H2AJ_HUMAN,sp\|Q8CGP5\|H2A1F_MOUSE,sp\|Q8CGP6\|H2A1H_MOUSE,sp\|Q8CGP7\|H2A1K_MOUSE,sp\|P22752\|H2A1_MOUSE,sp\|Q6GSS7\|H2A2A_MOUSE,sp\|Q64522\|H2A2B_MOUSE,sp\|Q64523\|H2A2C_MOUSE,sp\|Q8BFU2\|H2A3_MOUSE,sp\|Q8R1M2\|H2AJ_MOUSE | 2333.347 |  |
| 0.9257 | SS_20101013_QS_IDA_S_5_GluC.02834.02834.3 | 0.986847 | Jul-80 | V.n[89.01]TIAQGGVLPNIQAVLLPK[156.13]K[156.13]TE.S | sp\|Q96QV6\|H2A1A_HUMAN,sp\|P04908\|H2A1B_HUMAN,sp\|Q93077\|H2A1C_HUMAN,sp\|P20671\|H2A1D_HUMAN,sp\|Q96KK5\|H2A1H_HUMAN,sp\|Q99878\|H2A1J_HUMAN,sp\|P0C0S8\|H2A1_HUMAN,sp\|Q6FI13\|H2A2A_HUMAN,sp\|Q8IUE6\|H2A2B_HUMAN,sp\|Q16777\|H2A2C_HUMAN,sp\|Q7L7L0\|H2A3_HUMAN,sp\|Q9BTM1\|H2AJ_HUMAN,sp\|Q8CGP5\|H2A1F_MOUSE,sp\|Q8CGP6\|H2A1H_MOUSE,sp\|Q8CGP7\|H2A1K_MOUSE,sp\|P22752\|H2A1_MOUSE,sp\|Q6GSS7\|H2A2A_MOUSE,sp\|Q64522\|H2A2B_MOUSE,sp\|Q64523\|H2A2C_MOUSE,sp\|Q8BFU2\|H2A3_MOUSE,sp\|Q8R1M2\|H2AJ_MOUSE | 2333.347 |  |
| 0.9662 | SS_20101013_QS_IDA_S_5_GluC.02842.02842.3 | 0.993401 | Jul-80 | V.n[89.01]TIAQGGVLPNIQAVLLPK[156.13]K[156.13]TE.S | sp\|Q96QV6\|H2A1A_HUMAN,sp\|P04908\|H2A1B_HUMAN,sp\|Q93077\|H2A1C_HUMAN,sp\|P20671\|H2A1D_HUMAN,sp\|Q96KK5\|H2A1H_HUMAN,sp\|Q99878\|H2A1J_HUMAN,sp\|P0C0S8\|H2A1_HUMAN,sp\|Q6FI13\|H2A2A_HUMAN,sp\|Q8IUE6\|H2A2B_HUMAN,sp\|Q16777\|H2A2C_HUMAN,sp\|Q7L7L0\|H2A3_HUMAN,sp\|Q9BTM1\|H2AJ_HUMAN,sp\|Q8CGP5\|H2A1F_MOUSE,sp\|Q8CGP6\|H2A1H_MOUSE,sp\|Q8CGP7\|H2A1K_MOUSE,sp\|P22752\|H2A1_MOUSE,sp\|Q6GSS7\|H2A2A_MOUSE,sp\|Q64522\|H2A2B_MOUSE,sp\|Q64523\|H2A2C_MOUSE,sp\|Q8BFU2\|H2A3_MOUSE,sp\|Q8R1M2\|H2AJ_MOUSE | 2333.347 |  |
| 0.9926 | SS_20101013_QS_IDA_S_5_GluC.02949.02949.2 | 0.9967 | 31/36 | V.n[89.01]GQVGMAC[160.03]AISILGK[156.13]SLADE.L | sp\|P07195\|LDHB_HUMAN,sp\|P16125\|LDHB_MOUSE | 2034.979 |  |
| 0.9944 | SS_20101013_QS_IDA_S_5_GluC.02957.02957.2 | 0.997879 | 34/36 | V.n[89.01]GQVGMAC[160.03]AISILGK[156.13]SLADE.L | sp\|P07195\|LDHB_HUMAN,sp\|P16125\|LDHB_MOUSE | 2034.979 |  |
| 0.9889 | SS_20101013_QS_IDA_S_5_GluC.02965.02965.2 | 0.995078 | 32/36 | V.n[89.01]GQVGMAC[160.03]AISILGK[156.13]SLADE.L | sp\|P07195\|LDHB_HUMAN,sp\|P16125\|LDHB_MOUSE | 2034.979 |  |
| 0.9569 | SS_20101013_QS_IDA_S_5_GluC.03089.03089.2 | 0.95721 | 23/32 | I.n[89.01]NMLSLTQGLFRGLSRQE.T | sp\|P82675\|RT05_HUMAN | 2037.014 |  |
| 0.9369 | SS_20101013_QS_IDA_S_5_GluC.03096.03096.2 | 0.938476 | 23/32 | I.n[89.01]NMLSLTQGLFRGLSRQE.T | sp\|P82675\|RT05_HUMAN | 2037.014 |  |
| 0.9883 | SS_20101013_QS_IDA_S_5_GluC.03186.03186.2 | 0.98426 | 23/22 | E.n[89.01]IQTAVRLLLPGE.L | sp\|Q96A08\|H2B1A_HUMAN,sp\|P33778\|H2B1B_HUMAN,sp\|P62807\|H2B1C_HUMAN,sp\|P58876\|H2B1D_HUMAN,sp\|Q93079\|H2B1H_HUMAN,sp\|P06899\|H2B1J_HUMAN,sp\|O60814\|H2B1K_HUMAN,sp\|Q99880\|H2B1L_HUMAN,sp\|Q99879\|H2B1M_HUMAN,sp\|Q99877\|H2B1N_HUMAN,sp\|P23527\|H2B1O_HUMAN,sp\|Q16778\|H2B2E_HUMAN,sp\|Q5QNW6\|H2B2F_HUMAN,sp\|P57053\|H2BFS_HUMAN,sp\|P70696\|H2B1A_MOUSE,sp\|Q64475\|H2B1B_MOUSE,sp\|Q6ZWY9\|H2B1C_MOUSE,sp\|P10853\|H2B1F_MOUSE,sp\|Q64478\|H2B1H_MOUSE,sp\|Q8CGP1\|H2B1K_MOUSE,sp\|P10854\|H2B1M_MOUSE,sp\|Q8CGP2\|H2B1P_MOUSE,sp\|Q64525\|H2B2B_MOUSE | 1396.775 |  |
| 0.9846 | SS_20101013_QS_IDA_S_5_GluC.03266.03266.2 | 0.982988 | 24/24 | T.n[89.01]ALLDAAGVASLLT.T | sp\|P10809\|CH60_HUMAN,sp\|P63038\|CH60_MOUSE | 1301.69 |  |
| 0.9769 | SS_20101013_QS_IDA_S_5_GluC.03272.03272.2 | 0.97748 | 28/26 | T.n[89.01]ALLDAAGVASLLTT.A | sp\|P10809\|CH60_HUMAN,sp\|P63038\|CH60_MOUSE | 1402.738 |  |
| 0.9981 | SS_20101013_QS_IDA_S_5_GluC.03278.03278.2 | 0.998686 | Dec-30 | T.n[89.01]ALLDAAGVASLLTTAE.V | sp\|P10809\|CH60_HUMAN,sp\|P63038\|CH60_MOUSE | 1602.825 |  |
| 0.997 | SS_20101013_QS_IDA_S_5_GluC.03284.03284.2 | 0.997812 | 28/30 | T.n[89.01]ALLDAAGVASLLTTAE.V | sp\|P10809\|CH60_HUMAN,sp\|P63038\|CH60_MOUSE | 1602.818 |  |
|  |  |  |  |  |  |  |  |
|  |  |  |  |  |  |  |  |
