# Supplementary figures and images for: Unique Residues Involved in Activation of the Multitasking Protease/Chaperone HtrA from Chlamydia trachomatis
Source: PLoS One. 2011 Sep 8;6(9):e24547. doi: 10.1371/journal.pone.0024547 (PMC3169616; doi:10.1371/journal.pone.0024547)

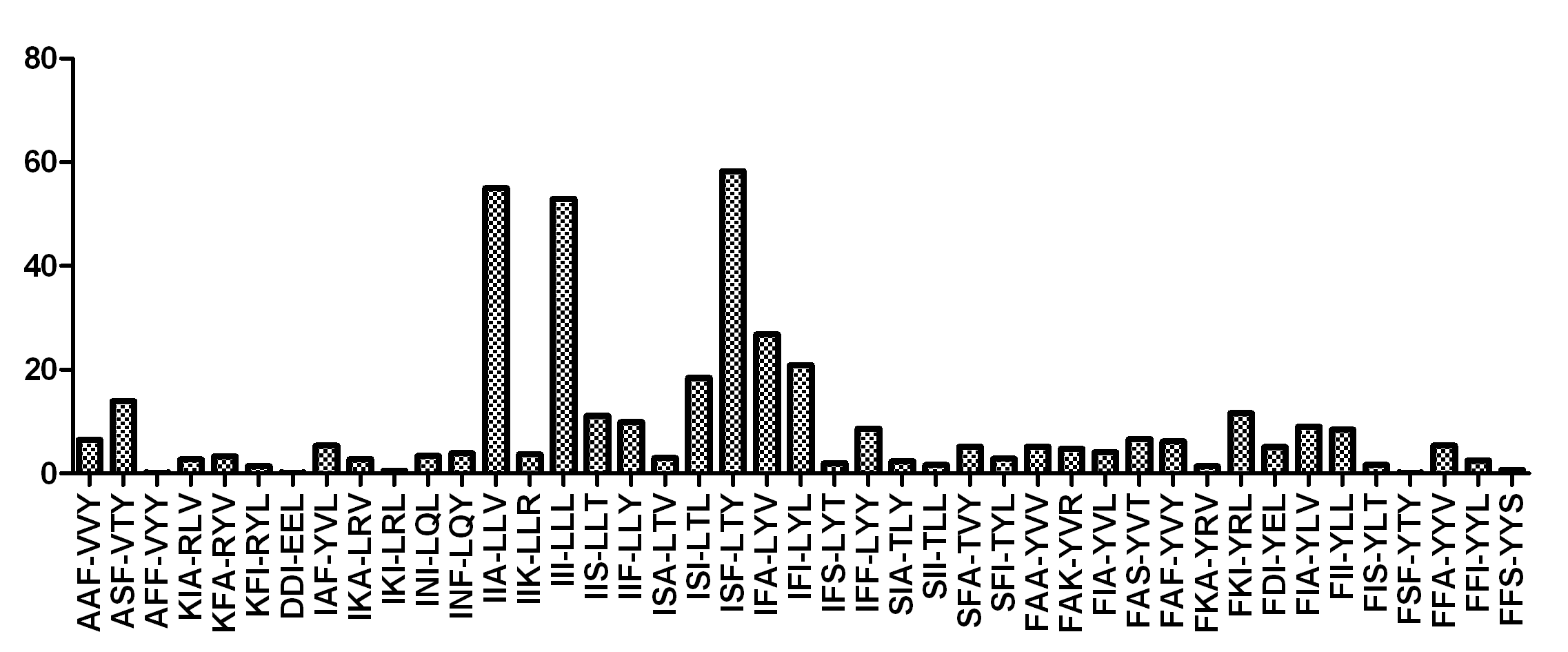


**Supporting information Fig S2.**

Supplement: Figure S2 — Results of protease assays using the REPLI Library all wells which has detectable substrates for CtHtrA are shown on the graph. The relative rate of fluorescent accumulation per min per ug of CtHtrA is shown on the Y axis. The well descriptions are indicated on the x axis. The wells contain 4–8 peptides with similar functional residues grouped, with a single residue variation between the two listed on each peptide. The peptides have a fluorophore (MCA) and quencher (Lys-DNP) with two trimers of glycine on each side of the variant trimer of amino acids in the middle (variant trimer ranges are indicated on the x axis). (DOCX) [file pone.0024547.s002.docx]

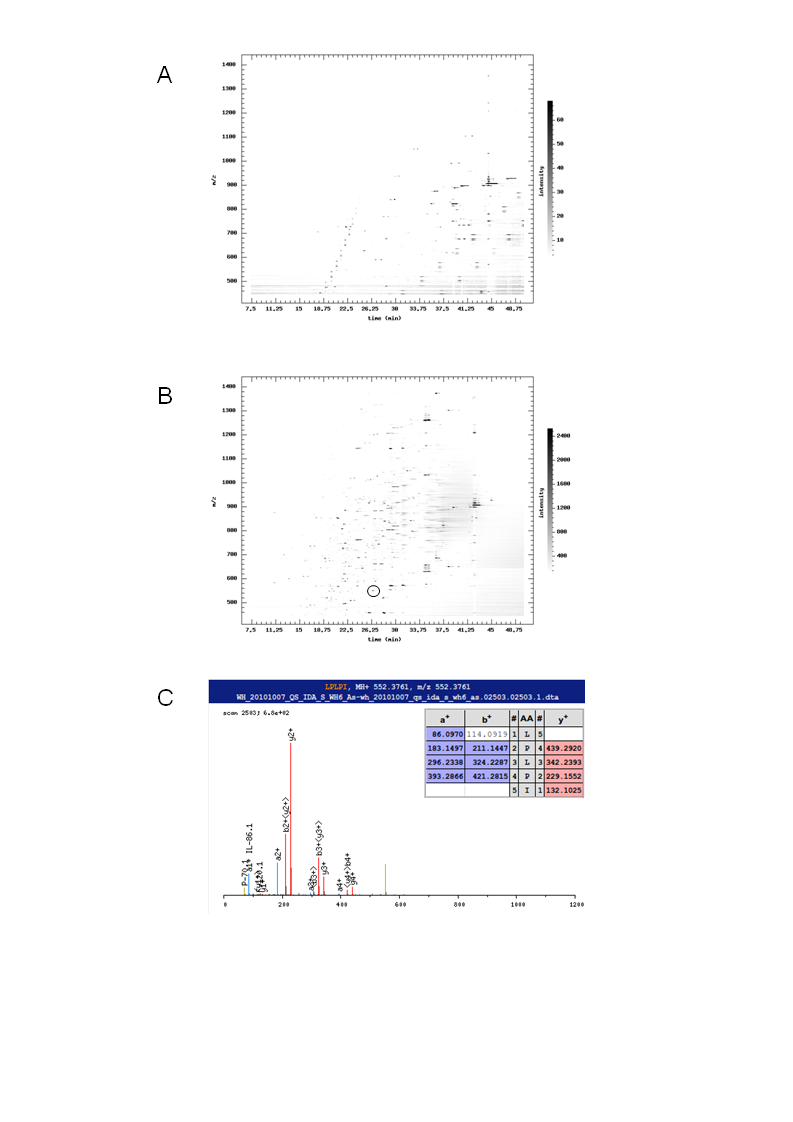


**Supporting information Fig S3. Characterisation of Bcas1 cleavage by CtHtrA.**

Supplement: Figure S3 — Characterisation of Bcas1 cleavage by CtHtrA. A. LC/MS plot of Bcas1 alone. B. B LC/MS plot of Bcas1 cleaved with CtHtrA shows addition of ions compared to Bcas1 alone. C. MS/MS of product ion 552.37 m/z, 26.3 min (circle in B) matches internal fragment of Bcas1. (DOCX) [file pone.0024547.s003.docx]
