# Supplementary material for: Unique Residues Involved in Activation of the Multitasking Protease/Chaperone HtrA from Chlamydia trachomatis
Source: PLoS One. 2011 Sep 8;6(9):e24547. doi: 10.1371/journal.pone.0024547 (PMC3169616; doi:10.1371/journal.pone.0024547)
Supplement: Figure S4 — Characterisation of protease specificity in the presence of the activator peptide using PICS. A. Web Logo summary of all peptide cleavage sites identified during PICS protocol with CtHtrA and Act1. B. The table below lists all peptide cleavage sites identified during PICS protocol with CtHtrA and Act1. (DOCX) [file pone.0024547.s004.docx]

**
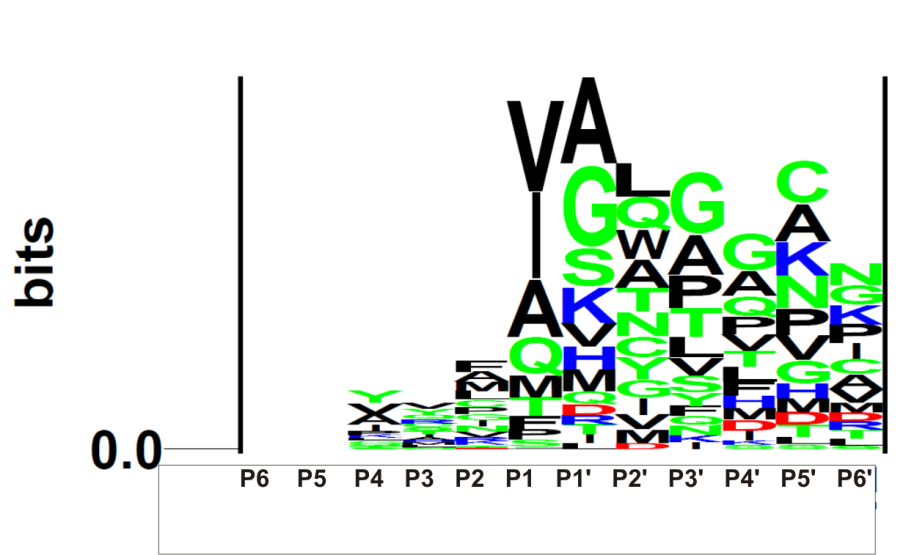
**

1. **WebLogo**

**Supporting information Fig S4A. Characterisation of protease specificity in the presence of the activator peptide using PICS.**

**Table S4B: PICs results of CtHtrA cleavages on GluC peptide library in presence of Act1.**

| **probability** | **ions** | **peptide** | **protein** | **calc_neutral_pep_mass** | |
| --- | --- | --- | --- | --- | --- |
| 0.980821 | 19/20 | I.n[89.01]AYGLDRTGK[156.13]GE.R | sp\|P08107\|HSP71_HUMAN,sp\|Q61696\|HS71A_MOUSE,sp\|P17879\|HS71B_MOUSE | 1281.6027 |  |
| 0.923957 | 18/20 | V.n[89.01]ALSTNTTK[156.13]VK[156.13]E.F | sp\|P06744\|G6PI_HUMAN | 1334.7117 |  |
| 0.996496 | 15/22 | V.n[89.01]GMGQK[156.13]DSYVGDE.A | sp\|P62736\|ACTA_HUMAN,sp\|P60709\|ACTB_HUMAN,sp\|P68032\|ACTC_HUMAN,sp\|P63261\|ACTG_HUMAN,sp\|P63267\|ACTH_HUMAN,sp\|P68133\|ACTS_HUMAN,sp\|P62737\|ACTA_MOUSE,sp\|P60710\|ACTB_MOUSE,sp\|P68033\|ACTC_MOUSE,sp\|P63260\|ACTG_MOUSE,sp\|P63268\|ACTH_MOUSE,sp\|P68134\|ACTS_MOUSE | 1400.5587 |  |
| 0.962301 | 19/24 | A.n[89.01]GLAGK[156.13]DPVQC[160.03]SRD.V | sp\|Q99497\|PARK7_HUMAN,sp\|Q99LX0\|PARK7_MOUSE | 1517.6967 |  |
| 0.857202 | 16/22 | D.n[89.01]MEVPVDGPVDM[147.04]Q.T | sp\|Q8BYK8\|ZC3H6_MOUSE | 1419.5717 |  |
| 0.743177 | 17/26 | E.n[89.01]QILDTTLSSPSSNA.P | sp\|Q8K3Z9\|PO121_MOUSE | 1520.7027 |  |
| 0.998728 | 21/30 | Q.n[89.01]ATVVAVGSGSK[156.13]GK[156.13]GGE.I | sp\|P61604\|CH10_HUMAN | 1546.8027 |  |
| 0.996075 | 15/30 | Q.n[89.01]ATVVAVGSGSK[156.13]GK[156.13]GGE.I | sp\|P61604\|CH10_HUMAN | 1546.8027 |  |
| 0.780935 | 16/14 | E.n[89.01]LLPAAC[160.03]LL.Q | sp\|Q53G59\|KLH12_HUMAN,sp\|Q8BZM0\|KLH12_MOUSE | 957.5027 |  |
| 0.881632 | 17/16 | V.n[89.01]GVFQHGK[156.13]VE.I | sp\|P34931\|HS71L_HUMAN,sp\|P08107\|HSP71_HUMAN,sp\|P54652\|HSP72_HUMAN,sp\|P11142\|HSP7C_HUMAN,sp\|Q61696\|HS71A_MOUSE,sp\|P17879\|HS71B_MOUSE,sp\|P16627\|HS71L_MOUSE,sp\|P17156\|HSP72_MOUSE,sp\|P63017\|HSP7C_MOUSE | 1115.5437 |  |
| 0.896892 | Apr-16 | V.n[89.01]GVFQHGK[156.13]VE.I | sp\|Q61696\|HS71A_MOUSE,sp\|P34931\|HS71L_HUMAN,sp\|P08107\|HSP71_HUMAN,sp\|P54652\|HSP72_HUMAN,sp\|P11142\|HSP7C_HUMAN,sp\|P17879\|HS71B_MOUSE,sp\|P16627\|HS71L_MOUSE,sp\|P17156\|HSP72_MOUSE,sp\|P63017\|HSP7C_MOUSE | 1115.5512 |  |
| 0.988929 | 22/30 | A.n[89.01]ATTAAPAAAAAPAK[156.13]VE.A | sp\|P05388\|RLA0_HUMAN | 1525.7807 |  |
| 0.998977 | 24/34 | V.n[89.01]AAATTAAPAAAAAPAK[156.13]VE.A | sp\|P05388\|RLA0_HUMAN | 1667.8557 |  |
| 0.906924 | 16/14 | I.n[89.01]GAYAK[156.13]ALE.I | sp\|Q99832\|TCPH_HUMAN,sp\|P80313\|TCPH_MOUSE | 937.4577 |  |
| 0.97376 | 20/20 | V.n[89.01]SLAVC[160.03]K[156.13]AGAVE.K | sp\|P06733\|ENOA_HUMAN,sp\|P17182\|ENOA_MOUSE | 1219.5937 |  |
| 0.897899 | Jul-26 | S.n[89.01]STGAAK[156.13]AVGK[156.13]VIPE.L | sp\|Q64467\|G3PT_MOUSE,sp\|P04406\|G3P_HUMAN,sp\|P16858\|G3P_MOUSE | 1470.8194 |  |
| 0.997698 | Jul-26 | S.n[89.01]STGAAK[156.13]AVGK[156.13]VIPE.L | sp\|Q64467\|G3PT_MOUSE,sp\|P04406\|G3P_HUMAN,sp\|P16858\|G3P_MOUSE | 1470.8194 |  |
| 0.998807 | Jul-26 | S.n[89.01]STGAAK[156.13]AVGK[156.13]VIPE.L | sp\|Q64467\|G3PT_MOUSE,sp\|P04406\|G3P_HUMAN,sp\|P16858\|G3P_MOUSE | 1470.8194 |  |
| 0.992627 | Jul-26 | S.n[89.01]STGAAK[156.13]AVGK[156.13]VIPE.L | sp\|Q64467\|G3PT_MOUSE,sp\|P04406\|G3P_HUMAN,sp\|P16858\|G3P_MOUSE | 1470.8194 |  |
| 0.98116 | Aug-26 | S.n[89.01]STGAAK[156.13]AVGK[156.13]VIPE.L | sp\|Q64467\|G3PT_MOUSE,sp\|P04406\|G3P_HUMAN,sp\|P16858\|G3P_MOUSE | 1470.8194 |  |
| 0.953051 | 22/24 | V.n[89.01]RAPMVNPTLGVHE.A | sp\|Q13423\|NNTM_HUMAN | 1507.7277 |  |
| 0.873014 | 16/20 | S.n[89.01]ANTFAITGHAE.A | sp\|Q96K17\|BT3L4_HUMAN,sp\|Q9CQH7\|BT3L4_MOUSE | 1218.5337 |  |
| 0.976902 | May-16 | Q.n[89.01]AVLLPK[156.13]K[156.13]TE.S | sp\|Q96QV6\|H2A1A_HUMAN,sp\|P04908\|H2A1B_HUMAN,sp\|Q93077\|H2A1C_HUMAN,sp\|P20671\|H2A1D_HUMAN,sp\|Q96KK5\|H2A1H_HUMAN,sp\|Q99878\|H2A1J_HUMAN,sp\|P0C0S8\|H2A1_HUMAN,sp\|Q6FI13\|H2A2A_HUMAN,sp\|Q8IUE6\|H2A2B_HUMAN,sp\|Q16777\|H2A2C_HUMAN,sp\|Q7L7L0\|H2A3_HUMAN,sp\|Q9BTM1\|H2AJ_HUMAN,sp\|Q8CGP5\|H2A1F_MOUSE,sp\|Q8CGP6\|H2A1H_MOUSE,sp\|Q8CGP7\|H2A1K_MOUSE,sp\|P22752\|H2A1_MOUSE,sp\|Q6GSS7\|H2A2A_MOUSE,sp\|Q64522\|H2A2B_MOUSE,sp\|Q64523\|H2A2C_MOUSE,sp\|Q8BFU2\|H2A3_MOUSE,sp\|Q8R1M2\|H2AJ_MOUSE | 1141.6859 |  |
| 0.927162 | 17/18 | L.n[89.01]AVLLSPLSRV.S | sp\|Q8IVV2\|LOXH1_HUMAN | 1141.6527 |  |
| 0.995311 | 17/16 | Q.n[89.01]AVLLPK[156.13]K[156.13]TE.S | sp\|Q96QV6\|H2A1A_HUMAN,sp\|P04908\|H2A1B_HUMAN,sp\|Q93077\|H2A1C_HUMAN,sp\|P20671\|H2A1D_HUMAN,sp\|Q96KK5\|H2A1H_HUMAN,sp\|Q99878\|H2A1J_HUMAN,sp\|P0C0S8\|H2A1_HUMAN,sp\|Q6FI13\|H2A2A_HUMAN,sp\|Q8IUE6\|H2A2B_HUMAN,sp\|Q16777\|H2A2C_HUMAN,sp\|Q7L7L0\|H2A3_HUMAN,sp\|Q9BTM1\|H2AJ_HUMAN,sp\|Q8CGP5\|H2A1F_MOUSE,sp\|Q8CGP6\|H2A1H_MOUSE,sp\|Q8CGP7\|H2A1K_MOUSE,sp\|P22752\|H2A1_MOUSE,sp\|Q6GSS7\|H2A2A_MOUSE,sp\|Q64522\|H2A2B_MOUSE,sp\|Q64523\|H2A2C_MOUSE,sp\|Q8BFU2\|H2A3_MOUSE,sp\|Q8R1M2\|H2AJ_MOUSE | 1141.6777 |  |
| 0.794165 | 21/24 | E.n[89.01]TIGVK[156.13]DGRGIITD.S | sp\|Q9BSQ5\|CCM2_HUMAN,sp\|Q8K2Y9\|CCM2_MOUSE | 1459.7707 |  |
| 0.992562 | 23/22 | I.n[89.01]GGIGTVPVGRVE.T | sp\|P68104\|EF1A1_HUMAN,sp\|Q05639\|EF1A2_HUMAN,sp\|Q5VTE0\|EF1A3_HUMAN,sp\|P10126\|EF1A1_MOUSE,sp\|P62631\|EF1A2_MOUSE | 1227.6277 |  |
| 0.770586 | 19/18 | I.n[89.01]K[156.13]LGLGIDEDE.V | sp\|P08238\|HS90B_HUMAN,sp\|P11499\|HS90B_MOUSE | 1203.5697 |  |
| 0.983567 | 20/34 | I.n[89.01]GDAAK[156.13]NQVALNPQNTVFD.A | sp\|P08107\|HSP71_HUMAN,sp\|Q61696\|HS71A_MOUSE,sp\|P17879\|HS71B_MOUSE | 2016.9577 |  |
| 0.989945 | 23/22 | I.n[89.01]VAPGK[156.13]GILAADE.S | sp\|P04075\|ALDOA_HUMAN,sp\|P09972\|ALDOC_HUMAN,sp\|P05064\|ALDOA_MOUSE,sp\|ALDOA_RABIT\| | 1255.6477 |  |
| 0.972036 | 30/34 | V.n[89.01]LLGPPGAGK[156.13]GTQAPRLAE.N | sp\|P54819\|KAD2_HUMAN | 1847.9927 |  |
| 0.743671 | Apr-30 | D.n[89.01]SAAAAFALDK[156.13]PALGPG.P | sp\|Q99811\|PRRX2_HUMAN | 1571.8096 |  |
| 0.817218 | 19/24 | I.n[89.01]GIPPAPRGVPQIE.V | sp\|P38646\|GRP75_HUMAN,sp\|P11021\|GRP78_HUMAN,sp\|P34931\|HS71L_HUMAN,sp\|P08107\|HSP71_HUMAN,sp\|P54652\|HSP72_HUMAN,sp\|P17066\|HSP76_HUMAN,sp\|P11142\|HSP7C_HUMAN,sp\|P38647\|GRP75_MOUSE,sp\|P20029\|GRP78_MOUSE,sp\|Q61696\|HS71A_MOUSE,sp\|P17879\|HS71B_MOUSE,sp\|P16627\|HS71L_MOUSE,sp\|P17156\|HSP72_MOUSE,sp\|P63017\|HSP7C_MOUSE | 1417.7387 |  |
| 0.999073 | 25/24 | T.n[89.01]GYPGDK[156.13]PVATMWE.S | sp\|SSPA_STAAU\| | 1565.6897 |  |
| 0.966445 | 29/32 | M.n[89.01]K[156.13]QTIGNSC[160.03]GTIGLIHAV.A | sp\|P09936\|UCHL1_HUMAN,sp\|Q9R0P9\|UCHL1_MOUSE | 1883.9597 |  |
| 0.991925 | 23/24 | T.n[89.01]GYPGDK[156.13]PVATMWE.S | sp\|SSPA_STAAU\| | 1565.6897 |  |
| 0.927162 | 21/36 | F.n[89.01]VGGLVGGPPGLAVGGAVGG.L | sp\|Q9NSK7\|CS012_HUMAN | 1577.8237 |  |
| 0.987188 | Aug-20 | A.n[89.01]K[156.13]LSDGVAVLK[156.13]V.G | sp\|P10809\|CH60_HUMAN,sp\|P63038\|CH60_MOUSE | 1271.7601 |  |
| 0.973903 | 20/18 | I.n[89.01]SILGK[156.13]SLADE.L | sp\|P07195\|LDHB_HUMAN,sp\|P16125\|LDHB_MOUSE | 1147.5795 |  |
| 0.988409 | Aug-20 | A.n[89.01]K[156.13]LSDGVAVLK[156.13]V.G | sp\|P10809\|CH60_HUMAN,sp\|P63038\|CH60_MOUSE | 1271.7601 |  |
| 0.907503 | 17/18 | I.n[89.01]SILGK[156.13]SLADE.L | sp\|P07195\|LDHB_HUMAN,sp\|P16125\|LDHB_MOUSE | 1147.5797 |  |
| 0.938928 | 20/22 | V.n[89.01]DTGIGMTK[156.13]ADLI.N | sp\|Q58FF8\|H90B2_HUMAN,sp\|Q58FF7\|H90B3_HUMAN,sp\|Q14568\|HS902_HUMAN,sp\|Q58FG1\|HS904_HUMAN,sp\|P07900\|HS90A_HUMAN,sp\|P08238\|HS90B_HUMAN,sp\|P07901\|HS90A_MOUSE,sp\|P11499\|HS90B_MOUSE | 1349.6567 |  |
| 0.954198 | 26/26 | V.n[89.01]ANLFNK[156.13]YPALTK[156.13]PE.N | sp\|P13797\|PLST_HUMAN,sp\|Q99K51\|PLST_MOUSE | 1748.9167 |  |
| 0.969521 | 23/26 | V.n[89.01]ANLFNK[156.13]YPALTK[156.13]PE.N | sp\|P13797\|PLST_HUMAN,sp\|Q99K51\|PLST_MOUSE | 1748.9167 |  |
| 0.826468 | 23/28 | I.n[89.01]ILNHPGQISAGYAPV.L | sp\|P68104\|EF1A1_HUMAN,sp\|Q5VTE0\|EF1A3_HUMAN,sp\|P10126\|EF1A1_MOUSE | 1623.8077 |  |
| 0.995475 | 27/32 | A.n[89.01]AVQAAILMGDK[156.13]SENVQD.L | sp\|P08107\|HSP71_HUMAN,sp\|Q61696\|HS71A_MOUSE,sp\|P17879\|HS71B_MOUSE | 1903.9017 |  |
| 0.943237 | 23/28 | I.n[89.01]ILNHPGQISAGYAPV.L | sp\|P68104\|EF1A1_HUMAN,sp\|Q5VTE0\|EF1A3_HUMAN,sp\|P10126\|EF1A1_MOUSE | 1623.8077 |  |
| 0.976002 | Jul-26 | A.n[89.01]ALK[156.13]K[156.13]ALAAAGYDVE.K | sp\|Q02539\|H11_HUMAN,sp\|P16403\|H12_HUMAN,sp\|P16402\|H13_HUMAN,sp\|P10412\|H14_HUMAN,sp\|P22492\|H1T_HUMAN,sp\|P15864\|H12_MOUSE,sp\|P43277\|H13_MOUSE,sp\|P43274\|H14_MOUSE,sp\|Q07133\|H1T_MOUSE | 1562.8456 |  |
| 0.995481 | 23/22 | I.n[89.01]AQGGVLPNIQAV.L | sp\|Q96QV6\|H2A1A_HUMAN,sp\|P04908\|H2A1B_HUMAN,sp\|Q93077\|H2A1C_HUMAN,sp\|P20671\|H2A1D_HUMAN,sp\|Q96KK5\|H2A1H_HUMAN,sp\|Q99878\|H2A1J_HUMAN,sp\|P0C0S8\|H2A1_HUMAN,sp\|Q6FI13\|H2A2A_HUMAN,sp\|Q8IUE6\|H2A2B_HUMAN,sp\|Q16777\|H2A2C_HUMAN,sp\|Q7L7L0\|H2A3_HUMAN,sp\|Q9BTM1\|H2AJ_HUMAN,sp\|P16104\|H2AX_HUMAN,sp\|Q8CGP5\|H2A1F_MOUSE,sp\|Q8CGP6\|H2A1H_MOUSE,sp\|Q8CGP7\|H2A1K_MOUSE,sp\|P22752\|H2A1_MOUSE,sp\|Q6GSS7\|H2A2A_MOUSE,sp\|Q64522\|H2A2B_MOUSE,sp\|Q64523\|H2A2C_MOUSE,sp\|Q8BFU2\|H2A3_MOUSE,sp\|Q8R1M2\|H2AJ_MOUSE,sp\|P27661\|H2AX_MOUSE | 1253.6437 |  |
| 0.910256 | Jun-26 | A.n[89.01]ALK[156.13]K[156.13]ALAAAGYDVE.K | sp\|Q02539\|H11_HUMAN,sp\|P16403\|H12_HUMAN,sp\|P16402\|H13_HUMAN,sp\|P10412\|H14_HUMAN,sp\|P22492\|H1T_HUMAN,sp\|P15864\|H12_MOUSE,sp\|P43277\|H13_MOUSE,sp\|P43274\|H14_MOUSE,sp\|Q07133\|H1T_MOUSE | 1562.8456 |  |
| 0.992891 | 22/22 | I.n[89.01]AQGGVLPNIQAV.L | sp\|Q96QV6\|H2A1A_HUMAN,sp\|P04908\|H2A1B_HUMAN,sp\|Q93077\|H2A1C_HUMAN,sp\|P20671\|H2A1D_HUMAN,sp\|Q96KK5\|H2A1H_HUMAN,sp\|Q99878\|H2A1J_HUMAN,sp\|P0C0S8\|H2A1_HUMAN,sp\|Q6FI13\|H2A2A_HUMAN,sp\|Q8IUE6\|H2A2B_HUMAN,sp\|Q16777\|H2A2C_HUMAN,sp\|Q7L7L0\|H2A3_HUMAN,sp\|Q9BTM1\|H2AJ_HUMAN,sp\|P16104\|H2AX_HUMAN,sp\|Q8CGP5\|H2A1F_MOUSE,sp\|Q8CGP6\|H2A1H_MOUSE,sp\|Q8CGP7\|H2A1K_MOUSE,sp\|P22752\|H2A1_MOUSE,sp\|Q6GSS7\|H2A2A_MOUSE,sp\|Q64522\|H2A2B_MOUSE,sp\|Q64523\|H2A2C_MOUSE,sp\|Q8BFU2\|H2A3_MOUSE,sp\|Q8R1M2\|H2AJ_MOUSE,sp\|P27661\|H2AX_MOUSE | 1253.6437 |  |
| 0.982855 | 16/18 | A.n[89.01]GQSVLLQLPQ.- | sp\|P35232\|PHB_HUMAN,sp\|P67778\|PHB_MOUSE | 1169.6117 |  |
| 0.99179 | 17/18 | A.n[89.01]GQSVLLQLPQ.- | sp\|P35232\|PHB_HUMAN,sp\|P67778\|PHB_MOUSE | 1169.6117 |  |
| 0.926604 | 28/32 | V.n[89.01]SNASC[160.03]TTNC[160.03]LAPLAK[156.13]VI.H | sp\|O14556\|G3PT_HUMAN,sp\|P04406\|G3P_HUMAN,sp\|Q64467\|G3PT_MOUSE,sp\|P16858\|G3P_MOUSE | 1934.9267 |  |
| 0.955722 | 25/26 | A.n[89.01]SC[160.03]TTNC[160.03]LAPLAK[156.13]VI.H | sp\|O14556\|G3PT_HUMAN,sp\|P04406\|G3P_HUMAN,sp\|Q64467\|G3PT_MOUSE,sp\|P16858\|G3P_MOUSE | 1662.8147 |  |
| 0.894982 | 22/22 | V.n[89.01]MAAASIANIVK[156.13]S.S | sp\|P17987\|TCPA_HUMAN,sp\|P11984\|TCPA1_MOUSE,sp\|P11983\|TCPA2_MOUSE | 1290.6677 |  |
| 0.943151 | Apr-72 | I.n[89.01]AQGGVLPNIQAVLLPK[156.13]K[156.13]TE.S | sp\|Q96QV6\|H2A1A_HUMAN,sp\|P04908\|H2A1B_HUMAN,sp\|Q93077\|H2A1C_HUMAN,sp\|P20671\|H2A1D_HUMAN,sp\|Q96KK5\|H2A1H_HUMAN,sp\|Q99878\|H2A1J_HUMAN,sp\|P0C0S8\|H2A1_HUMAN,sp\|Q6FI13\|H2A2A_HUMAN,sp\|Q8IUE6\|H2A2B_HUMAN,sp\|Q16777\|H2A2C_HUMAN,sp\|Q7L7L0\|H2A3_HUMAN,sp\|Q9BTM1\|H2AJ_HUMAN,sp\|Q8CGP5\|H2A1F_MOUSE,sp\|Q8CGP6\|H2A1H_MOUSE,sp\|Q8CGP7\|H2A1K_MOUSE,sp\|P22752\|H2A1_MOUSE,sp\|Q6GSS7\|H2A2A_MOUSE,sp\|Q64522\|H2A2B_MOUSE,sp\|Q64523\|H2A2C_MOUSE,sp\|Q8BFU2\|H2A3_MOUSE,sp\|Q8R1M2\|H2AJ_MOUSE | 2119.2153 |  |
| 0.884633 | 24/28 | I.n[89.01]HWGGVPNEFNGAVFI.N | sp\|SSPA_STAAU\| | 1730.7877 |  |
| 0.915637 | 25/28 | I.n[89.01]HWGGVPNEFNGAVFI.N | sp\|SSPA_STAAU\| | 1730.7877 |  |
| 0.986416 | Apr-68 | A.n[89.01]QGGVLPNIQAVLLPK[156.13]K[156.13]TE.S | sp\|Q96QV6\|H2A1A_HUMAN,sp\|P04908\|H2A1B_HUMAN,sp\|Q93077\|H2A1C_HUMAN,sp\|P20671\|H2A1D_HUMAN,sp\|Q96KK5\|H2A1H_HUMAN,sp\|Q99878\|H2A1J_HUMAN,sp\|P0C0S8\|H2A1_HUMAN,sp\|Q6FI13\|H2A2A_HUMAN,sp\|Q8IUE6\|H2A2B_HUMAN,sp\|Q16777\|H2A2C_HUMAN,sp\|Q7L7L0\|H2A3_HUMAN,sp\|Q9BTM1\|H2AJ_HUMAN,sp\|Q8CGP5\|H2A1F_MOUSE,sp\|Q8CGP6\|H2A1H_MOUSE,sp\|Q8CGP7\|H2A1K_MOUSE,sp\|P22752\|H2A1_MOUSE,sp\|Q6GSS7\|H2A2A_MOUSE,sp\|Q64522\|H2A2B_MOUSE,sp\|Q64523\|H2A2C_MOUSE,sp\|Q8BFU2\|H2A3_MOUSE,sp\|Q8R1M2\|H2AJ_MOUSE | 2048.1782 |  |
| 0.995028 | May-68 | A.n[89.01]QGGVLPNIQAVLLPK[156.13]K[156.13]TE.S | sp\|Q96QV6\|H2A1A_HUMAN,sp\|P04908\|H2A1B_HUMAN,sp\|Q93077\|H2A1C_HUMAN,sp\|P20671\|H2A1D_HUMAN,sp\|Q96KK5\|H2A1H_HUMAN,sp\|Q99878\|H2A1J_HUMAN,sp\|P0C0S8\|H2A1_HUMAN,sp\|Q6FI13\|H2A2A_HUMAN,sp\|Q8IUE6\|H2A2B_HUMAN,sp\|Q16777\|H2A2C_HUMAN,sp\|Q7L7L0\|H2A3_HUMAN,sp\|Q9BTM1\|H2AJ_HUMAN,sp\|Q8CGP5\|H2A1F_MOUSE,sp\|Q8CGP6\|H2A1H_MOUSE,sp\|Q8CGP7\|H2A1K_MOUSE,sp\|P22752\|H2A1_MOUSE,sp\|Q6GSS7\|H2A2A_MOUSE,sp\|Q64522\|H2A2B_MOUSE,sp\|Q64523\|H2A2C_MOUSE,sp\|Q8BFU2\|H2A3_MOUSE,sp\|Q8R1M2\|H2AJ_MOUSE | 2048.1782 |  |
| 0.98252 | Apr-68 | A.n[89.01]QGGVLPNIQAVLLPK[156.13]K[156.13]TE.S | sp\|Q96QV6\|H2A1A_HUMAN,sp\|P04908\|H2A1B_HUMAN,sp\|Q93077\|H2A1C_HUMAN,sp\|P20671\|H2A1D_HUMAN,sp\|Q96KK5\|H2A1H_HUMAN,sp\|Q99878\|H2A1J_HUMAN,sp\|P0C0S8\|H2A1_HUMAN,sp\|Q6FI13\|H2A2A_HUMAN,sp\|Q8IUE6\|H2A2B_HUMAN,sp\|Q16777\|H2A2C_HUMAN,sp\|Q7L7L0\|H2A3_HUMAN,sp\|Q9BTM1\|H2AJ_HUMAN,sp\|Q8CGP5\|H2A1F_MOUSE,sp\|Q8CGP6\|H2A1H_MOUSE,sp\|Q8CGP7\|H2A1K_MOUSE,sp\|P22752\|H2A1_MOUSE,sp\|Q6GSS7\|H2A2A_MOUSE,sp\|Q64522\|H2A2B_MOUSE,sp\|Q64523\|H2A2C_MOUSE,sp\|Q8BFU2\|H2A3_MOUSE,sp\|Q8R1M2\|H2AJ_MOUSE | 2048.1782 |  |
| 0.945977 | 24/32 | Q.n[89.01]GGVLPNIQAVLLPK[156.13]K[156.13]TE.S | sp\|Q96QV6\|H2A1A_HUMAN,sp\|P04908\|H2A1B_HUMAN,sp\|Q93077\|H2A1C_HUMAN,sp\|P20671\|H2A1D_HUMAN,sp\|Q96KK5\|H2A1H_HUMAN,sp\|Q99878\|H2A1J_HUMAN,sp\|P0C0S8\|H2A1_HUMAN,sp\|Q6FI13\|H2A2A_HUMAN,sp\|Q8IUE6\|H2A2B_HUMAN,sp\|Q16777\|H2A2C_HUMAN,sp\|Q7L7L0\|H2A3_HUMAN,sp\|Q9BTM1\|H2AJ_HUMAN,sp\|Q8CGP5\|H2A1F_MOUSE,sp\|Q8CGP6\|H2A1H_MOUSE,sp\|Q8CGP7\|H2A1K_MOUSE,sp\|P22752\|H2A1_MOUSE,sp\|Q6GSS7\|H2A2A_MOUSE,sp\|Q64522\|H2A2B_MOUSE,sp\|Q64523\|H2A2C_MOUSE,sp\|Q8BFU2\|H2A3_MOUSE,sp\|Q8R1M2\|H2AJ_MOUSE | 1920.1117 |  |
| 0.796584 | 20/32 | Q.n[89.01]GGVLPNIQAVLLPK[156.13]K[156.13]TE.S | sp\|Q96QV6\|H2A1A_HUMAN,sp\|P04908\|H2A1B_HUMAN,sp\|Q93077\|H2A1C_HUMAN,sp\|P20671\|H2A1D_HUMAN,sp\|Q96KK5\|H2A1H_HUMAN,sp\|Q99878\|H2A1J_HUMAN,sp\|P0C0S8\|H2A1_HUMAN,sp\|Q6FI13\|H2A2A_HUMAN,sp\|Q8IUE6\|H2A2B_HUMAN,sp\|Q16777\|H2A2C_HUMAN,sp\|Q7L7L0\|H2A3_HUMAN,sp\|Q9BTM1\|H2AJ_HUMAN,sp\|Q8CGP5\|H2A1F_MOUSE,sp\|Q8CGP6\|H2A1H_MOUSE,sp\|Q8CGP7\|H2A1K_MOUSE,sp\|P22752\|H2A1_MOUSE,sp\|Q6GSS7\|H2A2A_MOUSE,sp\|Q64522\|H2A2B_MOUSE,sp\|Q64523\|H2A2C_MOUSE,sp\|Q8BFU2\|H2A3_MOUSE,sp\|Q8R1M2\|H2AJ_MOUSE | 1920.1117 |  |
| 0.99418 | Jul-80 | V.n[89.01]TIAQGGVLPNIQAVLLPK[156.13]K[156.13]TE.S | sp\|Q96QV6\|H2A1A_HUMAN,sp\|P04908\|H2A1B_HUMAN,sp\|Q93077\|H2A1C_HUMAN,sp\|P20671\|H2A1D_HUMAN,sp\|Q96KK5\|H2A1H_HUMAN,sp\|Q99878\|H2A1J_HUMAN,sp\|P0C0S8\|H2A1_HUMAN,sp\|Q6FI13\|H2A2A_HUMAN,sp\|Q8IUE6\|H2A2B_HUMAN,sp\|Q16777\|H2A2C_HUMAN,sp\|Q7L7L0\|H2A3_HUMAN,sp\|Q9BTM1\|H2AJ_HUMAN,sp\|Q8CGP5\|H2A1F_MOUSE,sp\|Q8CGP6\|H2A1H_MOUSE,sp\|Q8CGP7\|H2A1K_MOUSE,sp\|P22752\|H2A1_MOUSE,sp\|Q6GSS7\|H2A2A_MOUSE,sp\|Q64522\|H2A2B_MOUSE,sp\|Q64523\|H2A2C_MOUSE,sp\|Q8BFU2\|H2A3_MOUSE,sp\|Q8R1M2\|H2AJ_MOUSE | 2333.3471 |  |
| 0.982968 | Jul-80 | V.n[89.01]TIAQGGVLPNIQAVLLPK[156.13]K[156.13]TE.S | sp\|Q96QV6\|H2A1A_HUMAN,sp\|P04908\|H2A1B_HUMAN,sp\|Q93077\|H2A1C_HUMAN,sp\|P20671\|H2A1D_HUMAN,sp\|Q96KK5\|H2A1H_HUMAN,sp\|Q99878\|H2A1J_HUMAN,sp\|P0C0S8\|H2A1_HUMAN,sp\|Q6FI13\|H2A2A_HUMAN,sp\|Q8IUE6\|H2A2B_HUMAN,sp\|Q16777\|H2A2C_HUMAN,sp\|Q7L7L0\|H2A3_HUMAN,sp\|Q9BTM1\|H2AJ_HUMAN,sp\|Q8CGP5\|H2A1F_MOUSE,sp\|Q8CGP6\|H2A1H_MOUSE,sp\|Q8CGP7\|H2A1K_MOUSE,sp\|P22752\|H2A1_MOUSE,sp\|Q6GSS7\|H2A2A_MOUSE,sp\|Q64522\|H2A2B_MOUSE,sp\|Q64523\|H2A2C_MOUSE,sp\|Q8BFU2\|H2A3_MOUSE,sp\|Q8R1M2\|H2AJ_MOUSE | 2333.3471 |  |
| 0.993457 | Jul-80 | V.n[89.01]TIAQGGVLPNIQAVLLPK[156.13]K[156.13]TE.S | sp\|Q96QV6\|H2A1A_HUMAN,sp\|P04908\|H2A1B_HUMAN,sp\|Q93077\|H2A1C_HUMAN,sp\|P20671\|H2A1D_HUMAN,sp\|Q96KK5\|H2A1H_HUMAN,sp\|Q99878\|H2A1J_HUMAN,sp\|P0C0S8\|H2A1_HUMAN,sp\|Q6FI13\|H2A2A_HUMAN,sp\|Q8IUE6\|H2A2B_HUMAN,sp\|Q16777\|H2A2C_HUMAN,sp\|Q7L7L0\|H2A3_HUMAN,sp\|Q9BTM1\|H2AJ_HUMAN,sp\|Q8CGP5\|H2A1F_MOUSE,sp\|Q8CGP6\|H2A1H_MOUSE,sp\|Q8CGP7\|H2A1K_MOUSE,sp\|P22752\|H2A1_MOUSE,sp\|Q6GSS7\|H2A2A_MOUSE,sp\|Q64522\|H2A2B_MOUSE,sp\|Q64523\|H2A2C_MOUSE,sp\|Q8BFU2\|H2A3_MOUSE,sp\|Q8R1M2\|H2AJ_MOUSE | 2333.3471 |  |
| 0.94343 | 24/28 | A.n[89.01]K[156.13]ALANVNIGSLIC[160.03]NV.G | sp\|P05386\|RLA1_HUMAN,sp\|P47955\|RLA1_MOUSE | 1700.8957 |  |
| 0.997743 | 32/36 | V.n[89.01]GQVGMAC[160.03]AISILGK[156.13]SLADE.L | sp\|P07195\|LDHB_HUMAN,sp\|P16125\|LDHB_MOUSE | 2034.9787 |  |
| 0.999342 | 29/36 | V.n[89.01]GQVGMAC[160.03]AISILGK[156.13]SLADE.L | sp\|P07195\|LDHB_HUMAN,sp\|P16125\|LDHB_MOUSE | 2034.9787 |  |
| 0.974293 | 21/36 | V.n[89.01]GQVGMAC[160.03]AISILGK[156.13]SLADE.L | sp\|P07195\|LDHB_HUMAN,sp\|P16125\|LDHB_MOUSE | 2034.9787 |  |
| 0.985551 | 23/36 | V.n[89.01]GQVGMAC[160.03]AISILGK[156.13]SLADE.L | sp\|P07195\|LDHB_HUMAN,sp\|P16125\|LDHB_MOUSE | 2034.9787 |  |
| 0.917842 | 18/22 | E.n[89.01]IQTAVRLLLPGE.L | sp\|Q96A08\|H2B1A_HUMAN,sp\|P33778\|H2B1B_HUMAN,sp\|P62807\|H2B1C_HUMAN,sp\|P58876\|H2B1D_HUMAN,sp\|Q93079\|H2B1H_HUMAN,sp\|P06899\|H2B1J_HUMAN,sp\|O60814\|H2B1K_HUMAN,sp\|Q99880\|H2B1L_HUMAN,sp\|Q99879\|H2B1M_HUMAN,sp\|Q99877\|H2B1N_HUMAN,sp\|P23527\|H2B1O_HUMAN,sp\|Q16778\|H2B2E_HUMAN,sp\|Q5QNW6\|H2B2F_HUMAN,sp\|P57053\|H2BFS_HUMAN,sp\|P70696\|H2B1A_MOUSE,sp\|Q64475\|H2B1B_MOUSE,sp\|Q6ZWY9\|H2B1C_MOUSE,sp\|P10853\|H2B1F_MOUSE,sp\|Q64478\|H2B1H_MOUSE,sp\|Q8CGP1\|H2B1K_MOUSE,sp\|P10854\|H2B1M_MOUSE,sp\|Q8CGP2\|H2B1P_MOUSE,sp\|Q64525\|H2B2B_MOUSE | 1396.7747 |  |
| 0.999999 | 25/24 | P.n[89.01]VLGPVRGPFPIIV.- | sp\|CASB_BOVIN\| | 1450.8367 |  |
| 0.999999 | 25/24 | P.n[89.01]VLGPVRGPFPIIV.- | sp\|CASB_BOVIN\| | 1450.8367 |  |
| 0.774111 | Nov-16 | A.n[89.01]AAAGAAGSA.A | sp\|Q96S94\|CCNL2_HUMAN | 733.3067 |  |
| 0.999999 | 26/24 | P.n[89.01]VLGPVRGPFPIIV.- | sp\|CASB_BOVIN\| | 1450.8367 |  |
| 0.999999 | 25/24 | P.n[89.01]VLGPVRGPFPIIV.- | sp\|CASB_BOVIN\| | 1450.8367 |  |
| 0.999999 | 24/24 | P.n[89.01]VLGPVRGPFPIIV.- | sp\|CASB_BOVIN\| | 1450.8367 |  |
| 0.999999 | 19/24 | P.n[89.01]VLGPVRGPFPIIV.- | sp\|CASB_BOVIN\| | 1450.8367 |  |
| 0.999999 | 23/24 | P.n[89.01]VLGPVRGPFPIIV.- | sp\|CASB_BOVIN\| | 1450.8367 |  |
| 0.999999 | 23/24 | P.n[89.01]VLGPVRGPFPIIV.- | sp\|CASB_BOVIN\| | 1450.8367 |  |
| 0.999999 | 22/24 | P.n[89.01]VLGPVRGPFPIIV.- | sp\|CASB_BOVIN\| | 1450.8367 |  |
| 0.999999 | 21/24 | P.n[89.01]VLGPVRGPFPIIV.- | sp\|CASB_BOVIN\| | 1450.8367 |  |
| 0.999999 | 20/24 | P.n[89.01]VLGPVRGPFPIIV.- | sp\|CASB_BOVIN\| | 1450.8367 |  |
| 0.999999 | 17/24 | P.n[89.01]VLGPVRGPFPIIV.- | sp\|CASB_BOVIN\| | 1450.8367 |  |
| 0.971462 | 23/26 | T.n[89.01]ALLDAAGVASLLTT.A | sp\|P10809\|CH60_HUMAN,sp\|P63038\|CH60_MOUSE | 1402.7377 |  |
| 0.999283 | 28/30 | T.n[89.01]ALLDAAGVASLLTTAE.V | sp\|P10809\|CH60_HUMAN,sp\|P63038\|CH60_MOUSE | 1602.8177 |  |
| 0.998888 | 29/30 | T.n[89.01]ALLDAAGVASLLTTAE.V | sp\|P10809\|CH60_HUMAN,sp\|P63038\|CH60_MOUSE | 1602.8177 |  |
|  |  |  |  |  |  |
