# Supplementary material for: Unique Residues Involved in Activation of the Multitasking Protease/Chaperone HtrA from Chlamydia trachomatis
Source: PLoS One. 2011 Sep 8;6(9):e24547. doi: 10.1371/journal.pone.0024547 (PMC3169616; doi:10.1371/journal.pone.0024547)
Supplement: Table S1 — Primers and amplification conditions used for site directed mutagenesis PCR. (DOCX) [file pone.0024547.s005.docx]

**Supporting information Table S1. Primers and amplification conditions used for site directed mutagenesis PCR**

| **Mutation** | **Primer sequence** | **PCR conditions** |
| --- | --- | --- |
| R299W | 5’-gtgatgggcaggtaacatggggctttttgggagttaccttg-3’ | Quickchange II |
|  | 5’-caaggtaactcccaaaaagccccatgttacctgcccatcac-3’ |  |
| L302N | 5’- caggtaacaagaggctttaacggagttaccttgcaaccg-3’ | Quickchange II |
|  | 5’-cggttgcaaggtaactccgttaaagcctcttgttacctg-3’ |  |
| I365D | 5’-gcgttgcgtaatgccgattccctaatgatgccaggg-3’ | Quickchange II |
|  | 5’-ccctggcatcattagggaatcggcattacgcaacgc-3’ |  |
| I265G | 5’-gccggtgtcagtggtagcgggg-3’ | Pfu 59°C anneal |
|  | 5’-agtattaaccccgataacttgaccattg-3’ |  |
| ΔPDZ1 | 5’-actgacaatggcagtattaacccc-3’ | Pfu 52°C anneal |
|  | 5’-tccctaatgatgccagggactcg-3’ |  |
| V266G | 5’-ccattggcagtggtagcggggg-3 | Pfu 60°C anneal |
|  | 5’-cagtattaaccccgataacttgaccattg-3’ |  |
| I242G | 5’ gccggtaatcctgggaattcaggc-3’ | Pfu 60°C anneal |
|  | 5’-agcatctgtttgaataaagtcttcg-3’ |  |
